# Structural and Biochemical Characterization of the Human Cyclophilin Family of Peptidyl-Prolyl Isomerases

**DOI:** 10.1371/journal.pbio.1000439

**Published:** 2010-07-27

**Authors:** Tara L. Davis, John R. Walker, Valérie Campagna-Slater, Patrick J. Finerty, Ragika Paramanathan, Galina Bernstein, Farrell MacKenzie, Wolfram Tempel, Hui Ouyang, Wen Hwa Lee, Elan Z. Eisenmesser, Sirano Dhe-Paganon

**Affiliations:** 1 Structural Genomics Consortium, University of Toronto, Toronto, Ontario, Canada; 2 Department of Physiology, University of Toronto, Toronto, Ontario, Canada; 3 University of Oxford, Headington, United Kingdom; 4 Department of Biochemistry & Molecular Genetics, University of Colorado Denver, Aurora, Colorado, United States of America; Brandeis University, United States of America

## Abstract

Peptidyl-prolyl isomerases catalyze the conversion between *cis* and *trans* isomers of proline. The cyclophilin family of peptidyl-prolyl isomerases is well known for being the target of the immunosuppressive drug cyclosporin, used to combat organ transplant rejection. There is great interest in both the substrate specificity of these enzymes and the design of isoform-selective ligands for them. However, the dearth of available data for individual family members inhibits attempts to design drug specificity; additionally, in order to define physiological functions for the cyclophilins, definitive isoform characterization is required. In the current study, enzymatic activity was assayed for 15 of the 17 human cyclophilin isomerase domains, and binding to the cyclosporin scaffold was tested. In order to rationalize the observed isoform diversity, the high-resolution crystallographic structures of seven cyclophilin domains were determined. These models, combined with seven previously solved cyclophilin isoforms, provide the basis for a family-wide structure∶function analysis. Detailed structural analysis of the human cyclophilin isomerase explains why cyclophilin activity against short peptides is correlated with an ability to ligate cyclosporin and why certain isoforms are not competent for either activity. In addition, we find that regions of the isomerase domain outside the proline-binding surface impart isoform specificity for both in vivo substrates and drug design. We hypothesize that there is a well-defined molecular surface corresponding to the substrate-binding S2 position that is a site of diversity in the cyclophilin family. Computational simulations of substrate binding in this region support our observations. Our data indicate that unique isoform determinants exist that may be exploited for development of selective ligands and suggest that the currently available small-molecule and peptide-based ligands for this class of enzyme are insufficient for isoform specificity.

**Enhanced version:**

**This article can also be viewed as an enhanced version in which the text of the article is integrated with interactive 3-D representations and animated transitions. Please note that a Web plugin is required to access this enhanced functionality. Instructions for the installation and use of the web plugin are available in Text S1.**

## Author Summary

Cyclophilins are proteins that catalyze the isomerization of prolines, interconverting this structurally important amino acid between *cis* and *trans* isomers. Although there are 17 cyclophilins in the human genome, the function of most cyclophilin isoforms is unknown. At least some members of this protein family are of interest for clinically relevant drug design, as they are targets of the drug cyclosporin, which is used as an immunosuppressant to treat patients following organ transplantation. The absence of a comprehensive picture of the similarities and differences between the different members of this protein family precludes effective and specific drug design, however. In the current study we undertake such a global structure∶function analysis. Using biochemical, structural, and computational methods we characterize the human cyclophilin family in detail and suggest that there is a previously overlooked region of these enzymes that contributes significantly to isoform diversity. We propose that this region may represent an important target for isoform-specific drug design.

## Introduction

Cyclophilins are peptidyl-prolyl isomerases (PPIases: EC 5.2.1.8) and are characterized by their ability to catalyze the interconversion of *cis* and *trans* isomers of proline [1]. Cyclophilins and the structurally unrelated FK506 binding proteins were initially described as the in vivo receptors for the natural products cyclosporin, FK506/tacrolimus, and rapamycin/sirolimus [2],[3]. The immunosuppressant effect of these natural products, while revolutionizing the field of organ transplantation, were eventually determined to be unrelated to the inherent isomerase activity of the PPIases [4]. However, these small molecules bind to the active site of PPIases with high affinity and are capable of blocking isomerase activity against peptide substrates, making them a useful tool for biochemical and cellular assays of PPIase function [5].

The physiological function of cyclophilin PPIase activity has been for many years described as a chaperone or foldase [6],[7]. Certainly this functionality is well documented, for instance in the maturation of steroid receptor complexes (along with Hsp90/Hsc70) [8] or in the interplay between NinaA and rhodopsin in *Drosophila* [9]. In addition, the isomerase activity of at least two cyclophilin isoforms is crucial for host∶virus interactions and for viral maturation processes, and this activity seems to be mediated through the PPIase active site [10],[11]. However, it has become increasingly apparent that isomerization of proline is not the sole function of the PPIases, with the first example being the nonimmunophilin Pin1, a PPIase of the parvulin type. Pin1 is able to catalyze isomerization of the proline bond for target substrates only when a serine or threonine preceding the target proline is phosphorylated [12]. This phosphorylation-dependent isomerization places Pin1 directly in the context of traditional signal transduction pathways, including those involved in cell proliferation and tumorigenesis [13]. The identification of Pin1 substrates revitalized the search for additional functions of the immunophilin-type PPIases; although there is no example of phosphorylation-dependent isomerization for either FK506 binding proteins or for cyclophilins, a subset of substrates for these types of PPIases are certainly also dependent on nonchaperone functions. PPIA, along with classical functions in the chaperone-mediated processes outlined above, interacts with the receptor tyrosine kinase Itk post-translationally and modulates the activity state of the already folded protein in vivo [14]. PPIA also is known to modulate HIV infectivity by interacting with a proline-containing sequence in the capsid protein Gag, also in the context of a well-folded protein module [15]. More recently, PPIA has been shown to interact with CD147 in a manner that is proline-dependent and mediated through the active site of the isomerase, but does not contribute to CD147 folding per se [16],[17]. In addition, both PPIA and the highly similar PPIB have been shown to interact with NS5B, an RNA-dependent RNA polymerase necessary for hepatitis C viral replication [10],[18]. The three other single-domain PPIases—which encode only the PPIase domain and, in the case of PPIB and PPIC, a signal sequence—and the 13 multidomain PPIases are less well characterized; most of what is known for these cyclophilins centers not on the isomerase active site but on distinct regions with no known enzymatic function. For instance, the single domain PPIase PPIH (SnuCyp20) participates in the spliceosome through interactions with the 60K component of the tri-snRNP, also known as hPRP4; however, the co-crystal structure of PPIH with a peptide derived from hPRP4 showed that this interaction was mediated exclusively through a face opposite that of the active site [19]. A similar situation was found in another spliceosomal cyclophilin, PPIL1, which interacts with the protein SKIP; NMR data indicate that the chemical shift perturbations in PPIL1 upon SKIP binding did not involve residues involved in proline turnover, and that binding to SKIP occurred even when PPIL1 was bound to cyclosporin A [20]. Finally, PPIE has an RNA-recognition motif (RRM) and has been reported to have RNA-specific isomerase activity [21].

Cyclophilins have been implicated in diverse signaling pathways, including mitochondrial apoptosis [22],[23], RNA splicing [24],[25], and adaptive immunity [26]. However, the proteins that are substrates for cyclophilins in these pathways have not been identified. Moreover, even basic questions concerning the biochemical properties of these enzymes have not been fully addressed. For instance, of the 17 annotated human cyclophilins only seven have been tested for isomerase activity or for the ability to bind cyclosporin [20],[27]–[32]. In vitro techniques aimed at delineating substrate specificity for the canonical family member PPIA have been only moderately successful; mutational analysis of short proline-containing motifs has found that PPIA is a very broadly specific enzyme [33],[34], despite the relatively small number of in vivo–validated substrates. In the case of phage display, the optimized binding sequence does not correspond to the substrate determinants that have been found in vivo for this isoform, and this sort of randomized screening has not been accomplished for any of the less ubiquitous isoforms [35]. Generally, the issue of in vitro versus in vivo substrate selectivity for the isomerases is problematic: for a given isomerase for which there is no knowledge of in vitro substrate specificity, it is difficult to find and validate in vivo substrates. Even for the isoforms that have been tested in vitro for their substrate preferences, there has been little or no correlation with later discovery of in vivo substrate sequences. Clues in some cases may be derived from the identity of other domains expressed in tandem with the cyclophilin domain; for instance, the RRM domain previously mentioned implies an RNA targeting function for PPIE and PPIL4, and likewise the U-box motif of PPIL2 implies involvement in ubiquitin conjugation pathways [36]. The WD-40 repeat of PPWD1 most likely confers a protein∶protein interaction function, as this is its main function in other systems; the same holds true for the TPR motifs of RanBP2 and PPID. However, useful comparisons of in vitro activity with in vivo physiology must wait until the cyclophilin family is more fully characterized with data from either or both lines of research.

In this study, we have screened 15 of the 17 human cyclophilins for their ability to catalyze proline isomerization against standard tetrapeptide proline motifs. We also have determined binding affinities for each cyclophilin family member for the natural product cyclosporin, and have determined the structures of seven PPIase domains to high resolution using X-ray crystallography. These extensive studies reveal interesting biochemical and enzymatic diversity that is consistent with structural data. The structures also provide an opportunity to assess the cyclophilin family for regions of diversity among all family members. In addition, in silico methods based on a family-wide structural analysis were used to characterize a molecular feature contiguous with the canonical active site that may account for substrate specificity. This new description of the cyclophilin peptidyl-prolyl isomerase family highlights regions of diversity that may prove crucial for future physiologically relevant substrate identification and chemical probe development.

## Results/Discussion

### Characterization of Cyclophilin Active Sites

**Figure 1:**
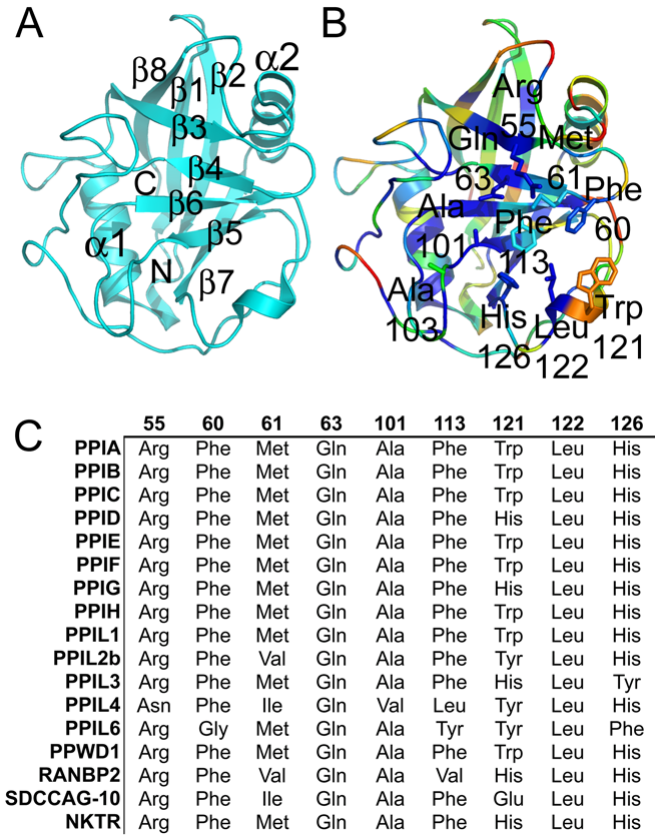
Structural elements of the cyclophilin fold and the definition of the active surface of PPIA. (A) Secondary structural elements of PPIA in ribbon representation, with key structural elements labeled. All structural outputs were generated using PyMol unless otherwise noted. (B) Consurf representation of sequence conservation within the human cyclophilin family; residues that compose the active surface of the cyclophilin family are labeled [81]. (C) Comparison of the sequences that define the active surface of the PPIase domain. Residue numbering corresponds to PPIA.

In order to elucidate the function of residues in the extended active site of the PPIase domain of the human cyclophilins, we probed the binding and catalytic function of these domains against either substrate or small-molecule inhibitors (see Figure 1 and Datapack S1 for graphical and tabular depictions of the active site). Three assays were utilized to explore these functions. In the first assay, changes in thermal stability were used to assess cyclosporin binding. This assay has been shown in several studies to be a reliable readout of small molecule binding for kinase and other enzyme families [37],[38]. Cyclosporin A (CsA) and the derivatives cyclosporin C, D, and H were screened against all PPIase domains except for PPIL3 and PPIL4, for which all constructs were insoluble or unstable in our hands (Table 1). Because of the inherent thermal stability characteristics of PPID and RanBP2, this technique was unable to distinguish between *apo* and cyclosporin-bound forms of those domains. However, data were collected for the remaining 13 isoforms, and binding to CsA, CsC, or CsD was noted for six isoforms published previously (PPIA, PPIB, PPIC, PPIE, PPIL1, and PPWD1) [20],[27]–[30],[32]. In addition, binding of CsA or derivatives was seen for PPIF, PPIG, PPIH, and NKTR. In the case of PPIG and PPIH, this explains previous data describing cyclosporin binding to the tri-snRNP complex that contains PPIH [25] and verifies the finding from a homolog that PPIG is capable of binding cyclosporin [39]. No binding was detected for PPIL2, PPIL6, or SDCCAG-10, making these, to our knowledge, the first set of human cyclophilins that have been found incompetent to ligate cyclosporin (Table 1). In order to quantify cyclosporin affinity we undertook isothermal calorimetry (ITC) analysis of all soluble cyclophilin isoforms; we found that a complete family-wide screen led to a range of binding affinities for CsA, expressed as the dissociation constant K_d_, from low nanomolar to near micromolar values. We were also able to confirm that under the experimental conditions we tested there was no evidence of CsA binding to PPIL2, PPIL6, or SDCCAG-10 (Table 1).

**Table 1:** Cyclosporin binding and tetrapeptide activity in the human cyclophilin family.

Cyclophilin	Assay		Thermal Stability		ITC
	Tetrapeptide Activity	Cyclosporin Binding	Basal T_agg_ (°C)	ΔT_agg_, CsC (°C)	K_d_, CsA (nM)
**PPIA**	yes	yes	45.9	1.9	6.8
**PPIB**	yes	yes	60.0	3.4	8.4*
**PPIC**	yes	yes	50.6	7.0	7.7*
**PPID**	yes	yes	n/a	n/a	61
**PPIE**	yes	yes	60.4	6.8	6.9
**PPIF**	yes	yes	52.4	10.7	6.7*
**PPIG**	yes	yes	55.6	2	51
**PPIH**	yes	yes	54.4	7.3	160
**PPIL1**	yes	yes	49.1	1.7	9.8*
**NKTR**	yes	yes	45.4	3.0	488
**PPWD1**	yes	yes	50.6	5.2	168
**PPIL2**	no	no	50.9	n/a	n/d
**PPIL6**	no	no	60.0	n/a	n/d
**RANBP2**	no	no	n/a	n/a	n/d
**SDCCAG-10**	no	no	44.8	n/a	n/d
**PPIL3**	not tested	n/a	n/a	n/a	n/a
**PPIL4**	not tested	n/a	n/a	n/a	n/a

Tetrapeptide activity is defined as the collapse of substrate *cis/trans* peaks in the presence of highly purified PPIase protein, as previously described [30],[40] and shown in Figure S1. Cyclosporin binding represents the combination of StarGazer and/or ITC data; briefly, any PPIase protein with a T_agg_ shift greater than 2°C in the presence of either cyclosporin A, C, or D is shown as a positive result. n/a indicates those cyclophilins that were either not tested or do not undergo a cooperative thermal transition [38],[85]. The basal T_agg_ is shown for all family members for whom a cooperative thermal transition was observed, along with the observed T_agg_ shift for one cyclosporin compound (CsC). “n/d” indicates no binding isotherm was noted in ITC under the experimental conditions outlined in this study.

*For ITC data, asterisks indicate the K_i_ values obtained in a recent study of isoform-selective inhibitors for six cyclophilins [67].

A two-dimensional NMR experiment (1H/1H TOCSY) described previously [30],[40], the only in vitro protease-free method available to probe for both substrate binding and catalytic activity of cyclophilins, was used to assess the commercially available tetrapeptides of sequence AAPF, AFPF, and AGPF [34]. The NMR-based assay confers the advantage of being a highly sensitive assay for the detection of substrate binding in addition to catalytic activity; the standard chymotrypsin-coupled assay can detect only catalysis and does not provide any direct measurement of binding [40]–[42]. A number of articles have documented the drawbacks of the protease-coupled assay [33],[42]–[44], an obvious example being that the addition of protease to the reaction mixture in the chymotrypsin-coupled assay requires additional testing to ensure that the enzymes and substrates being screened are not proteolytic targets [43]. Additionally, the NMR-based assay does not require substrates to contain chemical modifications, and can be used to measure effects of amino acid substitutions at regions distal to the target proline not measurable by other methods [42]. We detected binding and turnover for at least one of the tetrapeptide substrates tested for PPIA, PPIB, PPIC, PPID, PPIE, PPIF, PPIG, PPIH, PPIL1, PPWD1, and NKTR (Table 1; see Figure S1 for representative data showing binding and activity). This correlated well with previously determined activities [2],[20],[21],[28],[30],[45],[46], and established activity measurements for PPIF, PPIG, and NKTR. For all isoforms tested there was a strict correlation between the ability to bind cyclosporin and activity against the tetrapeptide substrates (Table 1).

**Figure 2:**
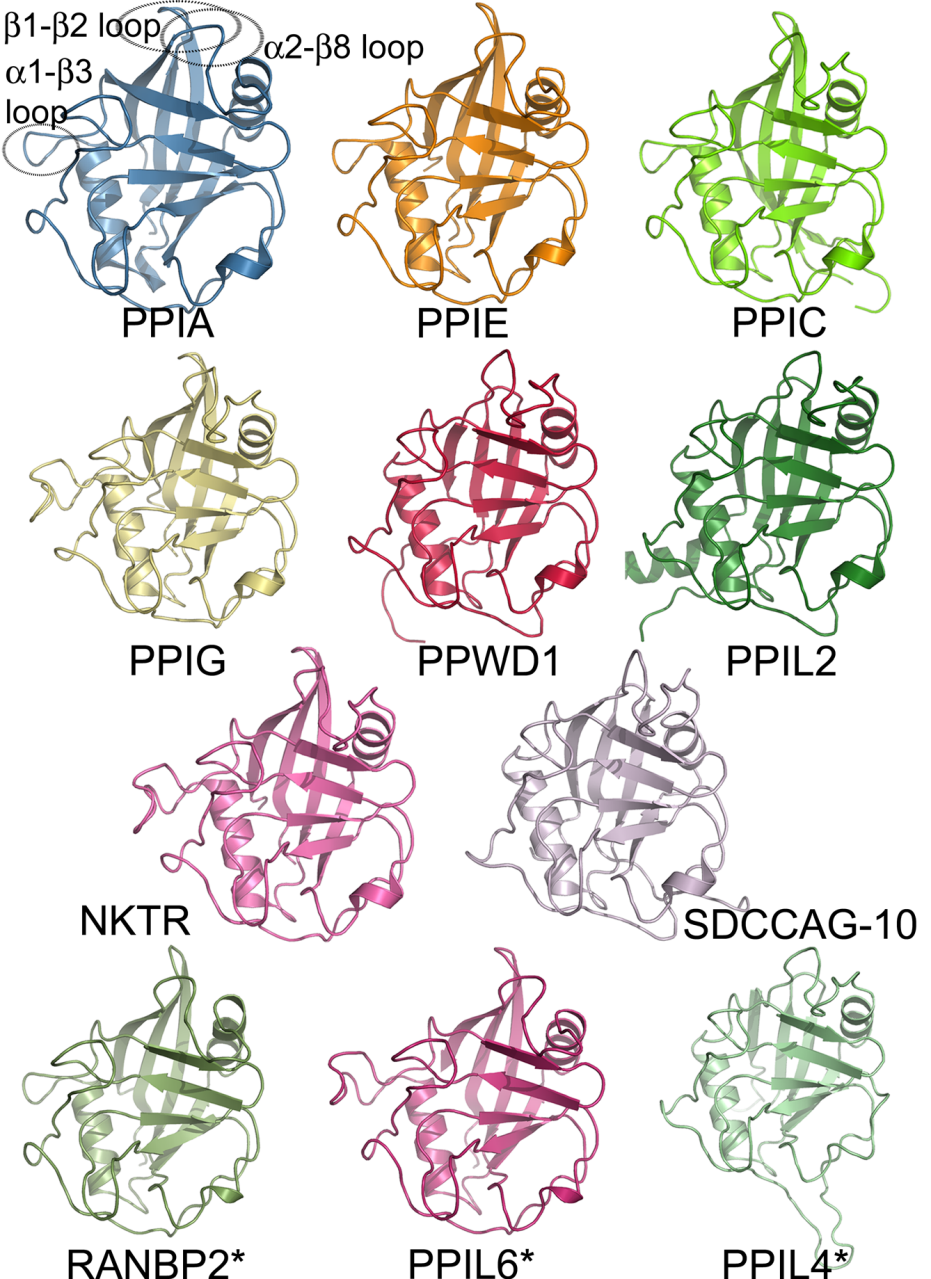
Structural coverage of the human cyclophilin family. Cartoon representation of the novel experimental and modeled structures of human cyclophilins associated with this manuscript. Only the isomerase domain is shown. The previously determined structure of PPIA is shown as a reference point, and loop regions discussed in the text are outlined with dotted ovals and labeled. The structures of RanBP2, PPIL6, and PPIL4 are marked with an asterisk, as they are derived from homology modeling using the Phyre server [47] and do not represent experimentally derived data. For crystallographic data concerning the structures shown here, refer to Table S1.

In order to understand the molecular basis of these results, we sought structural coverage of the entire human cyclophilin enzymatic class. We determined crystal structures of seven human PPIase domains—PPIC, PPIE, PPIG, PPWD1, PPIL2, NKTR, and SDCCAG-10 (Figure 2 and Datapack S1). There are six previously determined structures (PPIA, PPIB, PPIF, PPIH, PPIL1, and PPIL3). This leaves four structurally uncharacterized human PPIase domains of cyclophilins (PPID, PPIL4, PPIL6, and RanBP2) (Figure 2 and Table S1). However, if we include the highly homologous bovine structure for PPID (three amino acid substitutions compared to human) and compare the set of 14 isoforms for which we have experimental data, we find that they have very similar secondary structural elements (Figure 2). We can therefore use this dataset to provide excellent homology models for the remaining three isoforms (PPIL4, PPIL6, and the PPIase domain of RanBP2) (Figure 2). Models for these three isoforms were generated using the Phyre algorithm [47], and for all further discussions of the cyclophilin family the structures of all 17 PPIase domains will be considered.

All cyclophilins share a common fold architecture consisting of eight antiparallel β sheets and two α-helices that pack against the sheets (Figures 1 and 2). In addition, there is a short α-helical turn containing the active site residue Trp121 found in the β6-β7 loop region (Figure 1; all residue identities and numbers correspond to PPIA except where noted). RMSD across all atoms for all PPIase domains is less than 2 Å, and sequence identity over the same region varies from 61% to 86% (Figures 1, S2, and S3). The most divergent structures in this set are PPIL1, which is an NMR-derived structure (RMSD 1.7 Å), and the previously described PPWD1 (RMSD 1.4 Å) [30]. Excepting PPIL1 and PPWD1, the remaining experimental PPIase domains align over all atoms with RMSD ranging from 0.4 Å to 1.0 Å (see Figure 2 and also Figure S5 for a more detailed structural alignment). An overlay of the Phyre-derived modeled structures leads to an RMSD over all atoms of 1 Å or less compared to PPIA.

**Figure 3:**
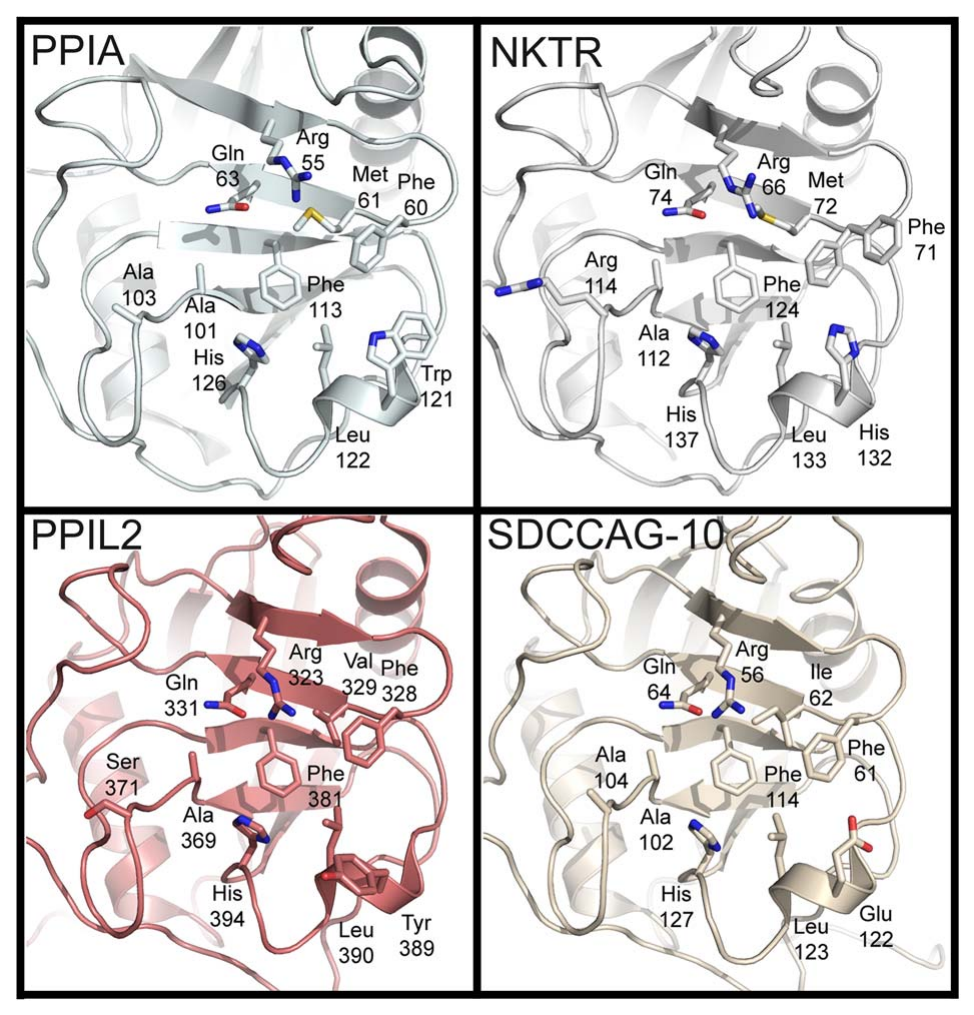
The structural consequences of substitutions in the cyclophilin active site. The residues described in Figure 1 are shown in stick representation for the divergent family members PPIA, NKTR, PPIL2, and SDCCAG-10. Note the orientation of the divergent residues Tyr389 in PPIL2 and Glu122 in SDCCAG-10 relative to Trp121 in PPIA or His132 in NKTR.

The active site of the cyclophilin family includes the invariant catalytic arginine (Arg55) and a highly conserved mixture of hydrophobic, aromatic, and polar residues including Phe60, Met61, Gln63, Ala101, Phe113, Trp121, Leu122, and His126 [48]–[50]. All of these sidechains contribute to an extensive binding surface along one face of the PPIase domain measuring roughly 10 Å along the Arg55–His126 axis and 15 Å along the Trp121–Ala101 axis (Figure 1). Many of these residues are well conserved across all PPIase domains and are thought to serve functions in either catalysis or substrate/inhibitor binding [48],[50],[51] (Figures 2, S2, and S3). Although there are sites of minor diversity among the family members at the Phe60, Met61, and His126 positions, the most striking correlation between cyclosporin binding, tetrapeptide identity, and active site residues is found at the Trp121 position. Our results clearly show that a tryptophan (as found in PPIA, PPIB, PPIC, PPIE, PPIF, PPIH, PPIL1, and PPWD1) or histidine (as found in PPID, PPIG, PPIL3, RANBP2, and NKTR) at this position is permissive for cyclosporin binding whilst other naturally occurring residues at this position (tyrosine in PPIL2, PPIL4, and PPIL6, and glutamic acid in SDCCAG10) abrogate cyclosporin binding under our experimental conditions (Table 1 and Figure 3). It has been shown that mutating Trp121 in PPIA to alanine or phenylalanine has a negative impact on cyclosporin affinity [51]–[53]. Mutation of the naturally occurring histidine in PPID to a tryptophan increases cyclosporin affinity dramatically, altering IC_50_ for cyclosporin from 1.9 mM to 28 nM and the K_d_^app^ to 12 nM [31],[54]. There are no mutational or computational data for the human cyclophilins that have a tyrosine or glutamic acid substitution at the Trp121 position; we therefore made a set of mutants to both PPIA (mutating Trp121 to either tyrosine or glutamic acid) and to PPIL2 (mutating Tyr389 to either tryptophan or histidine). As expected, mutation of Trp121 in PPIA to glutamic acid abolished activity of this protein; however, the tyrosine mutant retained the ability to catalyze proline isomerization, a novel result. More importantly, the single mutation of Tyr389 to tryptophan converted PPIL2 to an active isomerase, thereby illustrating the fundamental importance of this residue in conferring activity to the cyclophilin family (Figure S1B). However, the Tyr389 mutation to histidine did not lead to activity as measured by NMR under the experimental conditions assayed. For this reason, both the Tyr389 mutants were tested for CsA binding using ITC, and both the Tyr389Trp and Tyr389His mutants were found to bind CsA with micromolar affinity (1.6 µM and 6.6 µM for Trp and His respectively). Taken together, it is clear that there is some flexibility in the active site with regard to the Trp121 position: a tryptophan is clearly optimal at this position but tyrosine is somewhat permissive for activity, as is histidine. Glutamic acid at this position seems to be incompatible with isomerase activity.

Previous computational work with PPIA indicates that the function of Trp121 is mainly to serve to build a hydrophobic pocket for the substrate proline to insert (along with Phe60, Met61, Phe113, and Leu126) [55],[56]. However, our experimental data do not fully support this notion. To explain these results we modeled the interaction of CsA with the active site of cyclophilins, as the macrocyclic ring of cyclosporin structurally mimics the placement of the substrate residues N terminal and C terminal to the target proline (where the sequence Xaa-Pro-Yaa is denoted P1, P1′, and P2′ respectively) within the active site [48],[57]–[59]. Modeling of either CsA into the active site of a histidine containing isoform (like NKTR) or computational mutation of the Trp121 in a PPIA∶CsA complex structure indicated that similar hydrogen bond distances can exist between the indole moiety of tryptophan or the imidazole ring of histidine and the carbonyl of methylleucine 9 (MLE9) in CsA (Figure S4). Therefore either residue would be competent for binding, as we have shown experimentally. Conversely, a tyrosine modeled in the conformation to coordinate with CsA created a steric clash with the carbonyl of MLE9 (1.75 Å); in addition, there was a close steric conflict with the modeled Tyr residue and Cζ of the highly conserved Phe60 residue that helps form the proline-binding pocket (Figure S4). Perhaps this is why in our *apo* PPIL2 structure the tyrosine at this position pointed away from the active surface (Figure 2). Consistent with this, electron density for Phe71 residue in NKTR indicated that alternative conformations are possible for this residue, which may also explain why the PPIA Trp121Tyr mutant was still capable of coordinating substrate in vitro (Figure 2). We propose that the function of the residue at this position is to make a specific polar interaction with either the carbonyl of MLE9 in CsA or the carbonyl of a substrate peptide at the P2′ position (C terminal to the target proline).

Three cyclophilins neither bound cyclosporin nor tetrapeptide: PPIL2, PPIL6, and SDCCAG-10 (Table 1). It is clear that these three proteins are quite divergent in the active site compared to PPIA (Figure 1C). Perhaps more importantly they are, along with PPIL4, the only isoforms that substitute the residue Trp121 with a non-histidine residue. Additionally, PPIL4 does not possess the otherwise strictly conserved Arg55 (there is an asparagine at the equivalent position), so it is not surprising that this isoform does not show activity against standard substrates. The molecular function of the PPIase domain for these isoforms is unknown, but our structures suggest that these isoforms could still serve as proline-binding domains. Indeed, our assays show binding to the standard substrate suc-AGPF-pNA even where we do not detect isomerase activity (Figure S1A).

### Expanding the Definition of the Cyclophilin Active Site: The S2 Pocket and Gatekeeper Hypothesis

**Figure 4:**
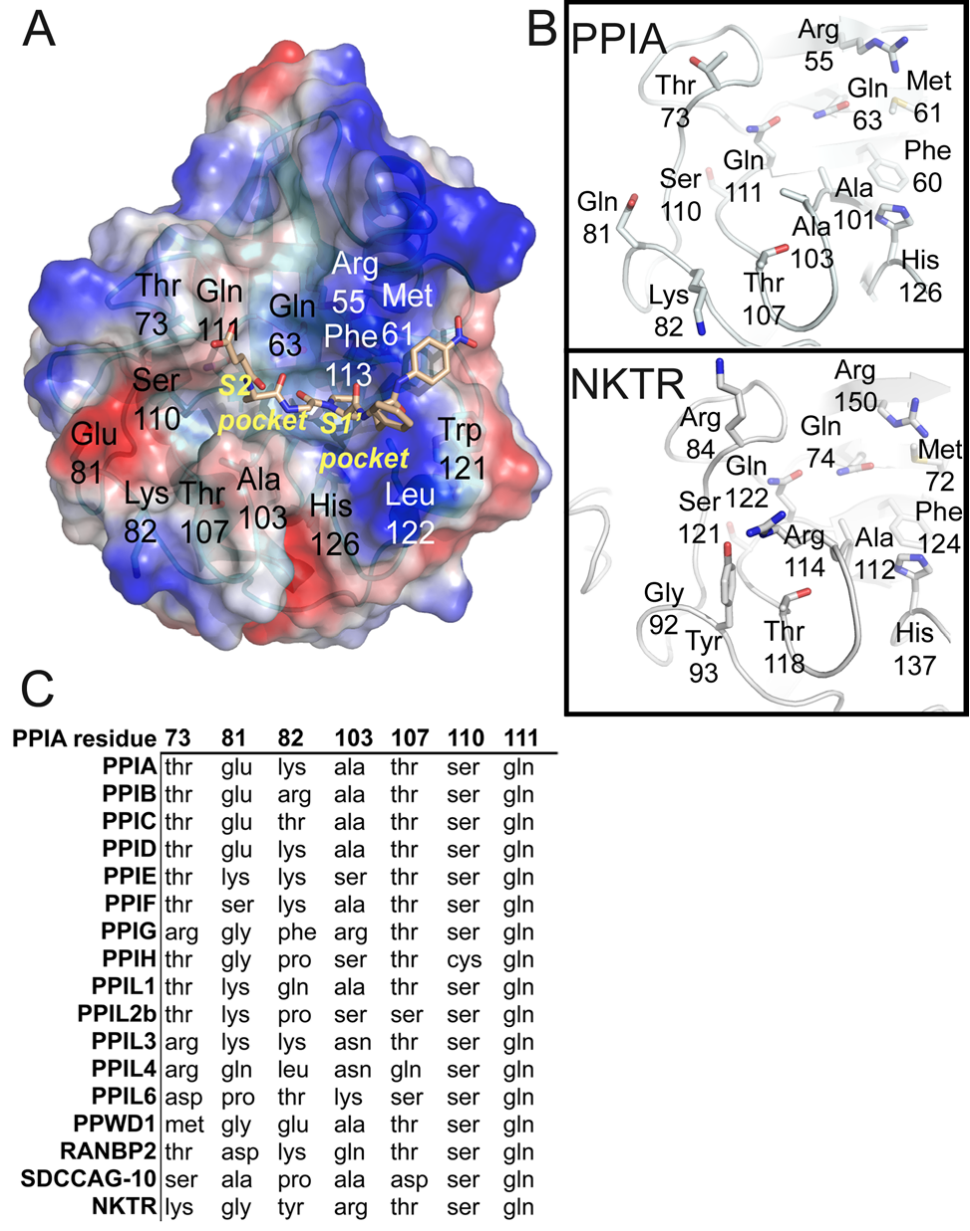
The S2 “gatekeeper” region of the human cyclophilins. (A) The definition of the S1′ pocket and S2 pocket is shown by depiction of a complex between PPIA and the tetrapeptide suc-AGPF-pNA (PDB 1ZKF). Surface representation of charges calculated within PyMol is shown, colored blue for basic and red for acidic regions. Residues around the active site and the S2 pocket are labeled according to PPIA numbering. (B) Sequence diversity of the gatekeeper residues in two PPIase domain structures. An “occluded” cyclophilin (NKTR) is shown in comparison to PPIA. As shown in Figure 5, these substitutions lead to diverse size and charge properties in this region of the cyclophilin active surfaces. (C) Comparison of the amino acids that define the S2 pocket of the PPIase domain. Residue numbering corresponds to PPIA.

**Figure 5:**
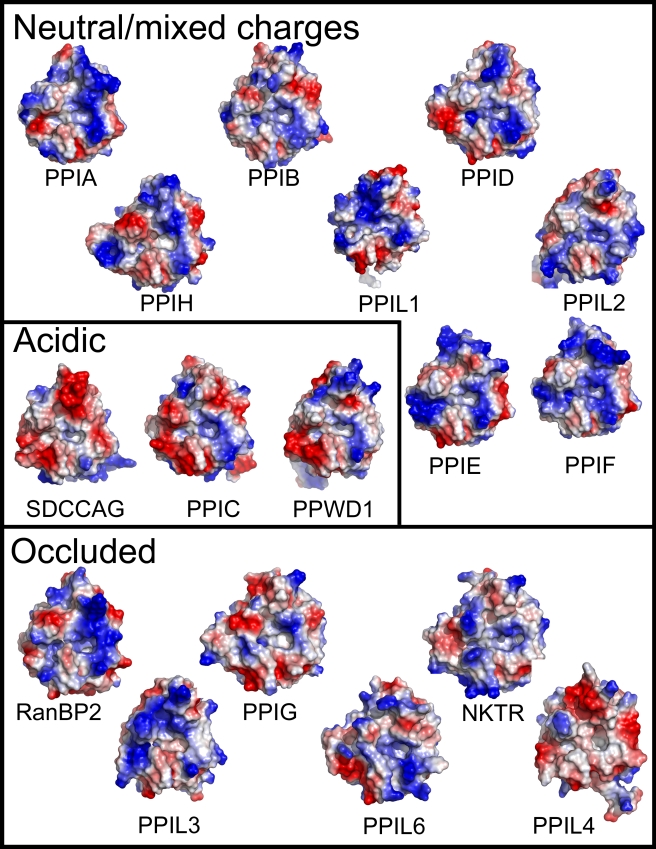
The diverse surfaces of the human cyclophilins. Surfaces of the human cyclophilins are shown colored by qualitative electrostatic potential. The scales of the potentials are all roughly the same (average potential: ±65 kBT/e) and range from ±56 kBT/e for PPIG to ±81kBT/e for PPIL4; all surfaces were calculated using the protein contact potential function in PyMol. As discussed in the text, the surfaces have been generally divided into those with neutral or mixed charge character surrounding the S2 pocket; those with largely acidic character around the S2 pocket; and those whose gatekeeper residue identities lead to occlusion of the S2 pocket.

PPIL2, PPIL6, and SDCCAG-10 are clearly divergent from the rest of the family in terms of in vitro activity. Next, a structural analysis of all family members was undertaken in order to probe for further isoform diversity. Examination of the surface of the PPIase domains near the active site revealed two pockets that potentially contribute to substrate specificity, binding, and turnover. The first pocket is the proline interaction surface (or S1′ pocket, where the target proline in substrate is again denoted as P1′) and is defined by the PPIA residues Phe113 at the base of the pocket and Phe60, Met61, Leu122, and His126 that form the sides of the pocket (Figure 4). As previously described, these residues are highly conserved across all PPIase isoforms and orthologs, consistent with minor discrimination against commercial substrates or cyclosporin [60]. The second pocket forms a surface that likely interacts with substrate residue P2 or P3 relative to the substrate proline, and so will be named the S2 pocket hereafter. Since the main-chain atoms of the β5-β6 loop define the base of the S2 pocket, the chemical identities of residues found in this region do not have much influence on the size and shape of the S2 pocket (Figure 4). Indeed, the S2 pocket is extremely uniform across cyclophilins; it is deep and relatively nonspecific, so it can accommodate long, short, polar, or hydrophobic sidechains without penalty. However, the S2 pocket surface is guarded by a set of “gatekeeper” residues whose sidechains are in a position to control access to this pocket. In PPIA, these residues are Thr73, Glu81, Lys82, Ala103, Thr107, Ser110, and Gln111 (Figure 4C). These gatekeeper residues at positions 81, 82, and 103 and the secondary gatekeeper at position 73 (so named because its position in most PPIase structures is pointed away from the S2 pocket) show major chemical and size variance. For instance, the residue that is at position 103 in PPIA varies from alanine in about half of the cyclophilin isoforms to a serine in PPIE, PPIH, and PPIL2; an arginine in PPIG and NKTR; lysine in PPIL6; asparagine in PPIL3 and PPIL4; and glutamine in RANBP2 (Figure 4C). The identities of the amino acids at positions 73, 81, and 82 are equally diverse across the cyclophilin family. The practical effect of this variance can be visualized by examining the surface properties of the cyclophilin family (Figure 5 and Datapack S1). These surfaces are clearly unique to the individual cyclophilin members, but can generally be classified into gatekeeper surfaces with mixed or neutral charges (see for example PPIA and several others); gatekeeper surfaces with overall acidic character (SDCCAG-10, PPIC, and PPWD1); and gatekeeper surfaces that occlude access to the S2 pocket (several; see Figure 5). The occluded set consists of the cyclophilin isoforms with bulky sidechains at the gatekeeper positions; for instance, NKTR has Lys84, Tyr93, and Arg114 compared to PPIA residues Thr73, Lys82, and Ala103 (Figures 4 and 5). Finally, residues within this region of PPIA, including Lys82, have previously been shown to be important for substrate binding as shown by NMR relaxation studies [61], consistent with a gatekeeper function.

### Structural Analysis of Regions Outside the Active Site

The S2 pocket is where conformational divergence throughout the cyclophilin family is greatest (Figure 2 and Datapack S1). Most of the remaining structural diversity is found in three of the loop regions connecting secondary structural elements. A subset of cyclophilins have a deletion in the β1-β2 loop region (residues Ala11-Pro16 in PPIA) that significantly alters the β sheet lengths in this region along with the loop between them. The division between “deleted” β1-β2 loops and “full-length” β1-β2 loops follows a phylogram distribution of PPIase domains, with the more conserved isoforms relative to PPIA (PPIB, PPIC, PPID, PPIE, PPIF, PPIG, PPIH, PPIL6, NKTR, and RanBP2) encoding full-length loops and the more divergent members by sequence (PPIL1, PPIL2, PPIL3, PPIL4, SDCCAG-10, and PPWD1) encoding deleted β1-β2 loops (Figure 2 and Figure S5). The α1-β3 loop (Thr41-Gly50) is also a region of structural diversity. There are three distinct classes of conformations adopted by this loop: the PPIA α1-β3 loop family, which includes PPIA, PPIB, PPIC, PPIE, and PPIF; a shorter version of the loop represented by the structures of PPIL1, PPIL2, PPIL3, PPIL4, SDCCAG-10, and PPWD1; and a longer version found in PPID, PPIG, PPIH, PPIL6, and NKTR. The short version of the α1-β3 loop changes the orientation of the α1 helix and the β3 sheet, and causes a ∼2 Å displacement of α1 relative to PPIA (Figure S5). Finally, the α2-β8 loop (Gly146-Lys155) has two distinct groups: the standard conformation found in PPIA, PPIE, PPIF, PPIL6, and RANBP2, and the conformation adopted by all other isoforms (Figure S5). Interestingly, two regions found to have structural divergence (the β1-β2 and α2-β8 loops) form a contiguous surface on the “back” face of the cyclophilin fold relative to the active site. Sequence and structural diversity in this region could indicate a preference for different potential binding partners, as the back face of cyclophilins has previously been shown to mediate protein∶protein interactions [19],[20]. However, it seems that for substrate interactions mediated by the proline-binding pocket isoform selectivity is likely to be determined by the S2 pocket region rather than these distal regions. Thus, the functional significance of the S2 pocket will be further explored with regard to its effect on substrate binding and specificity.

### Cyclophilin Diversity in the “S2 Gatekeeper Region”

Our biochemical data are the latest evidence that molecular determinants for tetrapeptide substrate or cyclosporin binding may not be identical to molecular determinants for physiologically relevant substrates, and supplements other recent publications along these lines [62],[63]. Additionally, structural analysis suggests that the region surrounding the S2 pocket is an attractive target to design isoform specificity. As commercially available ligands and substrates are unable to effectively probe this region of the cyclophilin family, we turned to in silico techniques to obtain insight into isoform gatekeeper identity and its relationship to accessibility to the S2 pocket. Four hundred test peptides of the general form Xaa-Zaa-Gly-Pro (corresponding to substrate positions P3-P2-P1-P1′) were docked into a subset of cyclophilin family members (PPIA, PPIL2, PPIC, PPWD1, and NKTR). These proteins were chosen because of the diversity of the amino acids in the gatekeeper and S2 pocket regions (Figure 5). Monte Carlo simulations were performed to sample conformational space for each combination of cyclophilin isoform and test peptide, allowing flexibility of the P2 and P3 residues of the potential substrate and of the sidechains of the gatekeepers at positions comparable to PPIA Thr73 (gatekeeper 1), Lys82 (gatekeeper 2), and Ala103 (gatekeeper 3) while keeping the rest of the protein rigid [64]. The sidechain of Arg377 in PPIL2, which is a glycine in the other cyclophilins investigated, was also allowed flexibility as it contributes a unique chemistry to the S2 region. Throughout the Monte Carlo simulations (200,000 iterations) tethers were imposed on the Gly and Pro residues to ensure that the tetrapeptides would remain bound to the active site. We made an assumption, based on a number of previous crystallographic and NMR-based studies of the cyclophilins, that the position and coordination of the Gly-Pro sequence of substrate is relatively fixed within the active site of the PPIase. Several structural studies with both synthetic and natural substrate data bound to PPIA support this assumption [30],[50],[59]. It was computationally necessary to fix the P1 and P1′ positions upon the enzyme in order to allow for more degrees of freedom at the P2 and P3 positions in our simulations; without these tethers we would have been testing the contribution of these two residues to the overall ability of substrate to bind the entire active site. While this is a very interesting line of study the interaction of proline in the proline binding or P1′ pocket was not the focus of the current work. For each combination of cyclophilin isoform and tetrapeptide, the lowest-energy complex was chosen as the preferred conformation of the bound complex, and an estimate of the binding energy was calculated using ICM [65]. Additionally, since low-energy complexes may or may not include significant interactions at the S2 pocket, the distance between the tetrapeptide and the Cα of the gatekeeper equivalent to PPIA Lys82 was calculated. This metric was designed to query for tetrapeptides that both bind with favorable energy in the S2 pocket, and also fill the S2 pocket if possible.

**Figure 6:**
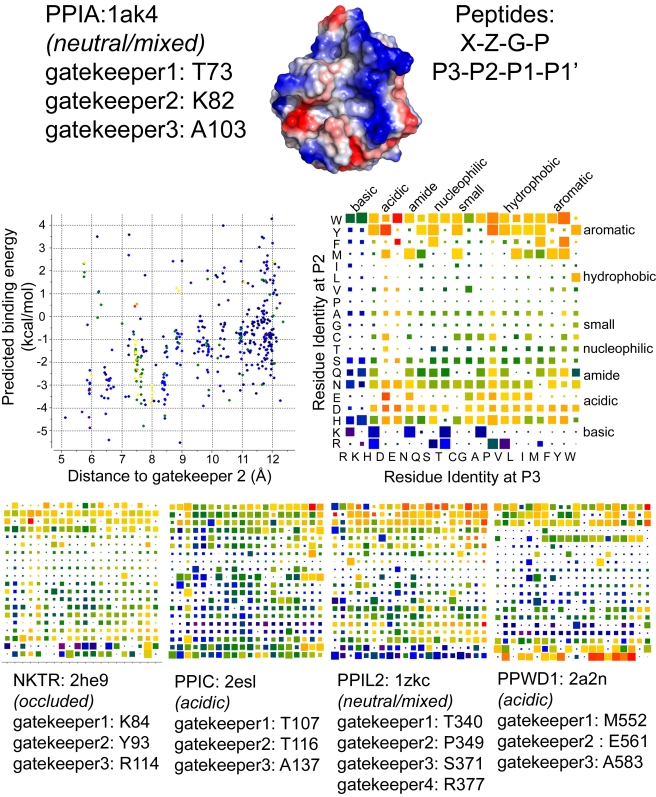
Peptide:protein simulations for five members of the cyclophilin family. At the top are detailed results for PPIA. Simulations were set up with the structure of PPIA (PDB 1AK4) and with 400 peptides corresponding to the sequences X-Z-G-P, where X and Z are each of the possible combinations of the naturally occurring 20 amino acids. Gatekeeper residues were allowed flexibility during the simulations and are noted for each family member. The middle two panels are graphical representations of the PPIA results. On the left is a scatter plot with the energy metric on the y-axis and the distance metric on the x-axis. The lower left quadrant is where the highest-scoring peptide combinations are plotted (greatest negative energy and closest interaction with the S2 pocket). The color of each spot in the plot corresponds to the hydrogen bonding potential between that particular peptide and PPIA, with red indicating greater values (nine for the PPIA simulation) and purple indicating lesser values (two for the PPIA simulation). Scatter plots for all other simulations are found in Figure S6. In the panel on the right, the identity of the residue at position P3 is plotted along the x-axis, and the identity of the residue at the P2 position is plotted along the y-axis. The general chemical classification for each residue set is indicated. At the intersection of each x, y point is a square representing the binding energy and distance metrics. Red indicates greater binding energy for that x, y pair; purple indicates lesser energy. Value ranges for PPIA were −5.5 (red) to 4.3 (purple). Larger squares fill the S2 pocket to a greater extent. The bottom four panels are x, y arrays for four other cyclophilin simulations. Coloring and axes are as in the middle right panel. Note that the energy value ranges for the five x, y arrays are not identical and are as follows: NKTR (red = −7.0, purple = 5.0), PPIC (red = −5.9, purple = 1.3), PPIL2 (red = −4.7, purple = 1.9), PPWD1 (red = −7.0, purple = 1.0).

An energetic preference for aromatics interacting with the S2 pocket was found for PPIA, in particular tryptophan or tyrosine (Figure 6; for scatter plot representation see Figure S6). In addition, there were a few peptides containing methionine, lysine, or arginine at the P2 position that extended deeply into the S2 pocket, albeit with poor predicted binding energies. Peptides with isoleucine, leucine, valine, proline, alanine, glycine, cysteine, threonine, or serine at the P2 position were disfavored, with poor predicted binding energies. We observed much less discrimination for the identity of the P3 position, although there is a clear selection against basic chemistries (Figure 6). Visual inspection of the top 10 model complexes predicted for PPIA based on the energy metric (EFGP, EWGP, DYGP, DEGP, DDGP, YWGP, PYGP, EDGP, YFGP, and PWGP) showed that all of the residues at the P2 position are well positioned to fill the S2 pocket of PPIA, while inspection of some models that scored poorly (RFGP, ERGP, DFGP) showed incomplete entry into the S2 pocket. In addition, these models indicated interactions between the residue at the P3 position and the gatekeeper 1 residue, or with the P1′ pocket and the key active site residue Arg55.

The published data on specificity for PPIA are consistent with our findings. Previous in vitro phage display experiments with PPIA (designed to probe substrate preferences at the P1 to P8′ positions) found a strong preference for phenylalanine at the P2 and glutamic acid at the P3 position; these residues were provided by the expression vector used in the phage display and therefore biased the pool of samples available for initial selection [35]. Substitution of this glutamic acid/phenylalanine series with any other residues, however, lessened the signal on an array, thereby confirming a preference for these chemistries in solution. Our simulations support this chemical preference for acidic residues at P3 followed by aromatic residues at P2 (Figure 6). A well-characterized substrate in vivo for PPIA is the HIV capsid; there are several sequence variants that have been studied both in solution and in crystallographic experiments, and all sequences have either methionine or alanine at the P2 position and histidine or alanine at the P3 position [50],[66]. In the structures of PPIA with these peptides, the alanine does not fill the S2 pocket, and this is likely the reason why it does not score well in our modeling trials. Neither histidine nor alanine at the P3 position is predicted to score highly by our modeling trials, and in the co-crystal structures these residues are not making any significant contacts to the gatekeeper 1 region of PPIA. The validated in vivo substrate CD147 was also investigated. The natural sequence that is acted upon by PPIA is ALWP, which was not predicted to bind tightly to PPIA based on either the phage display data or our simulations, and experimentally was found to have rather weak affinity [17]. Finally, the PPIA substrate Itk contains the targeted sequence ENNP, which is a relatively high-scoring P3 and P2 sequence combination based on our models [14]. Our simulations recapitulate the experimental data that is available, but imply that none of the in vitro or in vivo substrates studied to date for PPIA interact with the S2 pocket with optimized space-filling or energetic properties.

In order to begin experimental validation of our in silico predictions, a peptide “test set” composed of the following sequences was synthesized: DEGPF, DFGPF, DYGPF, YGGPF, and VRGPF. We then monitored catalysis of all of these potential substrates using our NMR-based assay (Figure S1). These peptides were selected in order to allow us to discriminate between cyclophilin isoforms; initial studies were conducted with PPIA in order to optimize experimental conditions for the detection of binding and catalysis. Our data indicated that, although PPIA was competent to bind all five peptides, only those predicted to have significant scores on the binding energy metric were substrates for proline isomerization (DEGPF, DFGPF, and DYGPF; see Figure 6 and Figure S1). The two peptides that were not efficient substrates for catalysis (YGGPF and VRGPF) both yielded poor predicted binding energies in our docking study to PPIA. That there was little discrimination with our NMR assay between DEGPF, DFGPF, and DYGPF was somewhat inconsistent with our simulations, as the model peptide for DFGP did not extend fully into the S2 pocket. It is possible that while tethering the P1 and P1′ Gly-Pro sequence allowed us to obtain a large number of reasonable structures at the P2 and P3 positions, it may have artificially increased our in silico binding affinity in a way that we cannot recapitulate in vitro. It is also possible that this spatial constraint upon our simulations biased our results towards substrates with the key interacting residue at the P2 position. Perhaps in vitro it is the P3 position that contributes significantly to binding energy; therefore the binding contributed by the aspartic acid in the current test set was the significant determinant for binding to PPIA in addition to the identity of the residue at the P2 position. Regardless, these experimental results will allow us to next analyze the capacity of our test set to discriminate among cyclophilin isoforms. Additionally, as all of our test peptides are identical at the P1, P1′, and P2′ positions, we can see for the first time that substitutions at amino acids in the P2 and P3 positions have measurable effects on the ability of the broad specificity enzyme PPIA to bind and catalyze proline containing sequences.

Distinct patterns of chemical preference were noted for PPIC, PPIL2, NKTR, and PPWD1 (Figure 6; for scatter plot representation see Figure S6). Much like PPIA, the PPIase domains of PPIC and PPIL2 showed an energetic preference for tryptophan at the P2 position; and for PPIL2 and NKTR isoleucine, leucine, valine, proline, alanine, glycine, cysteine, threonine, and serine at the P2 position resulted in poor predicted binding energies and little penetration into the S2 pocket (Figure 6). Indeed, for NKTR there were relatively few tetrapeptide combinations with both favorable predicted binding energy and penetration into the S2 pocket; this is easily rationalized by the extremely narrow gap between the gatekeeper 1 and gatekeeper 3 regions in the NKTR structure, which occlude the S2 pocket and restrict the types of residues that can stably associate with the pocket without steric or charge clashes (Figures 5, 6). PPIC showed a distinct preference pattern for aromatic residues at P2 preceded by basic or aromatic residues at P3 (Figure 6). This is most likely due to the substitution of gatekeeper 2 and the overall acidic character of this region of PPIC relative to PPIA (Figure 5).

In the case of PPIL2, there was near equivalency between the aromatics at position P2, with perhaps a slight energetic preference for tryptophan but strong affinities for tyrosine and phenylalanine as well. Likewise there was little discrimination at the P3 position (Figure 6). Compared to PPIL2 simulations, the results for PPWD1 were striking: the acidic surface characteristics of this isoform selected strongly for an arginine at the P2 position, while lysine and aromatic residues also yielded good predicted binding energies (Figures 5 and 6). Of the surfaces tested, only PPWD1 provided a surface where strong energy scores were measured for basic residues at this position. Experimentally, the construct used initially for crystallization of PPWD1 contained a sequence AEGP found N-terminal to the PPIase domain, and this sequence was found associated with a neighboring PPIase domain in the crystal structure. NMR-based assays showed that AEGP bound PPWD1 but was not a good substrate for the enzyme, which correlates well with the poor binding energy predicted for the AEGP tetrapeptide in our simulations [30]. Again, the scarcity of experimental data for cyclophilin isoforms limits the ability to validate the simulations; but to the extent that such information exists, it correlates well with our in silico findings. Current efforts are underway to measure binding and/or proline isomerization of our test set peptides with NKTR, PPIC, PPIL2, and PPWD1; we predict based on our above analysis that several of our test set peptides would bind well to most or all of our test cyclophilins (see DYGP and DFGP in Figure 6), while others could be selective for some isoforms over others (VRGP, which has good energy metrics for PPWD1 but not for any other isoform in the current study). Although in vitro validation of our in silico results are still ongoing, we believe that the initial data we present here provide the basis for a renewed study of the S2 pocket of the human cyclophilins as a potential locus of chemical and substrate diversity.

In conclusion, there are cyclophilin family members that, while sharing overall conservation with active members of the family, do not possess isomerase activity in our assays. For PPIL2 and SDCCAG-10, both of which have been found associated with spliceosomal complexes, it may be that it is the non-active surface of the PPIase domain that performs the major function as in the cases of PPIH and PPIL1. Additionally, it may well be that the function of the PPIase domain in these cyclophilins is to simply bind proline-containing motifs. Our NMR data suggest this option, as binding without measurable catalysis to proline sequences is observed for all isoforms we were able to test.

Chemical probes such as cyclosporin are unselective with regard to the cyclophilin family (Table 1) [67]. Although a recent report focusing on aryl 1-indanylketones showed binding to PPIA, PPIF, and PPIL1 while not binding to PPIB, PPIC, or PPIH [67], it seems that any ligand that coordinates exclusively with the S1′ pocket and/or Trp121 region is unlikely to be selective with respect to the entire cyclophilin family. Potentially, the S2′ or S3′ region of the isomerase domain could be a site of selectivity; it is clear from our surface representations (Figure 5) that this is a variable part of the cyclophilin domain. However, our results indicate that a clear virtual chemical fingerprint exists for the S2 and S3 positions of the isomerase domain. For instance, PPIA and PPWD1 seem to have restricted sets of sidechains that are preferred at the P2 position (and the P3 position in the case of PPIA), while PPIC appears to be more promiscuous. The highly occluded nature for the S2 pocket exhibited by NKTR results in a restrictive set of allowed tetrapeptide sequences for this isoform; several other isoforms in the cyclophilin family also exhibit this type of gatekeeper restriction. Because of the very distinct molecular features of the S2 region, both in terms of the highly “druggable” S2 pocket and the chemical diversity seen for the gatekeeper residues, targeting this region of the cyclophilins for pharmacophore design and selection is more likely to result in tight binders with greater specificity for particular isoforms in the family.

## Materials and Methods

### Cloning, Expression, and Purification of Isomerase Domains

Detailed materials and methods for cloning, expression, purification, and crystallization of all novel isomerase domain structures solved as part of the Structural Genomics Consortium are freely available at the Web site http://www.sgc.utoronto.ca/; where methods differ significantly from the following they are noted for each isoform in Text S2. In general, full-length cDNA clones were obtained from the Mammalian Gene Collection (accession numbers noted below). Constructs based around the predicted isomerase domain boundaries were cloned into pET28a using ligation-independent cloning methods (LIC) (BD Biosciences, San Jose, CA, USA) and transformed into BL21 Gold DE3 cells (Stratagene, La Jolla, CA, USA). The resulting vectors encode an N-terminal His_6_ tag with a thrombin cleavage site. Mutants of cyclophilin constructs were created either using standard Quickchange protocols (Stratagene) or by LIC-based methods on PCR fused gene products. Cultures were grown in Terrific Broth medium at 37°C to OD_600_ of 6 and induced at 15°C overnight with the addition of 50–100 µm isopropyl thio-β-D-galactoside (IPTG). Pellets were resuspended in 20 mL of lysis buffer (50 mm Tris, pH 8.0, 500 mm NaCl, 1 mm phenylmethanesulfonyl fluoride and 0.1 mL of general protease inhibitor (P2714, Sigma, St. Louis, MO, USA) and lysed by sonication; lysates were then centrifuged for 20 min at 69,673*g*. The supernatant was loaded onto nickel nitrilotriacetic acid resin (Qiagen, Valencia, CA, USA), washed with five column volumes of lysis buffer and five column volumes of low imidazole buffer (lysis buffer+10 mm imidazole, pH 8), and eluted in 10 mL of elution buffer (lysis buffer+250 mm imidazole, pH 8, and 10% glycerol). If the His_6_ tag was cleaved for crystallization purposes, then one unit of thrombin (Sigma) per milligram of protein was added to remove the tag overnight at 4°C. For gel filtration, a column packed with HiLoad Superdex 200 resin (GE Healthcare, Piscataway, NJ, USA) was pre-equilibrated with gel filtration buffer (lysis buffer+5 mM β-mercaptoethanol and 1 mM ethylenediaminetetraacetic acid). Peak fractions were pooled and concentrated using Amicon concentrators (10,000 molecular mass cut-off; Millipore, Danvers, MA, USA). The protein was generally used at 250–500 µM for crystallization screening.

### Crystallization and Structure Solution of Isomerase Domains

Generally, crystal hits were initially prepared in sitting drop 96-well format. Proteins were set up as 1 µL protein+1 µL reservoir solution and incubated at 18°C for 24 h to 1 mo. If crystal optimization was required it was performed in 24-well hanging drop format with 1 µL protein+1 µL reservoir solution. Crystals were cryoprotected with mother liquor with 10%–15% glycerol. Datasets were collected on an in-house FR-E SuperBright Cu rotating anode/Raxis IV++ detector (Rigaku Americas, The Woodlands, TX, USA); except for PPIC, which was collected at APS 19-BM. Data was integrated and scaled using the HKL2000 program package [68],[69]. The program PHASER [70] was used as part of the CCP4 suite [71] to find the molecular replacement solution. Manual rebuilding was performed using either O [72] or COOT [73], and refined using REFMAC [74] in the CCP4I program suite [75]. In most cases ARP/wARP was utilized to assist in model building and iterative refinement of starting phases [76]. Final models were evaluated using PROCHECK [77] and MOLPROBITY [78], with all models judged to have excellent stereochemistry and no residues in disallowed regions of Ramachandran space.

#### PPIC

Specifically, optimized PPIC crystals were obtained using the hanging drop vapor diffusion method. Crystals grew when the protein (encoding residues at 15 mg/mL was preincubated with cyclosporin A in a 1∶2 molar ratio for at least overnight and then mixed with the reservoir solution in a 1∶1 volume ratio. The drop was equilibrated against a reservoir solution containing 25% PEG MME 550, 0.1 M zinc acetate, 0.1 M MES at pH 6.5.

#### PPIE

Diffracting crystals leading to the structure grew when the protein was mixed at 20 mg/mL with the reservoir solution (containing 34% PEG 8K, 0.2 M NH_4_SO_4_, and 0.1 M bis-Tris, pH 6) in a 1∶1 volume ratio.

#### PPIG

Purified PPIG K125A/E126A (indicating mutations at the indicated residues) was crystallized using the sitting drop vapor diffusion method at 18°C by mixing 0.2 µl of the protein solution with 0.2 µl of the reservoir solution containing 2 M NH_4_SO_4_, 0.2 M NaCl, 0.1 M Hepes, pH 7.5.

#### PPIL2

Diffracting crystals leading to the structure grew when the protein was mixed at 20 mg/mL with the reservoir solution (containing 0.8 M KNa-tartrate, 0.1 M Hepes, pH 7.5) in a 1∶1 volume ratio.

#### PPWD1

Purified PPWD1 was crystallized using the hanging drop vapor diffusion method. Crystals grew when the protein (12 mg/mL) was mixed with the reservoir solution in a 1∶1 volume ratio, and the drop was equilibrated against a reservoir solution containing 1.7 M NH_4_SO_4_, 0.1 M Na-cacodylate, 0.2 M Na-acetate, pH 5.7. Full methods can be found in [30].

#### NKTR

Crystals grew in hanging drop format when protein at 15 mg/mL was mixed with reservoir containing 21% PEG 3350, 0.25 M KSO_4_ in a 1∶1 ratio.

#### SDCCAG-10

Crystals were obtained when the protein at 20 mg/mL was mixed with reservoir solution containing 20% PEG 3350 and 0.2 M NaI in 1∶1 ratio in hanging drop format.

### Thermal Stabilization Assay

All protein samples used for static light scattering (StarGazer) trials were assessed for purity utilizing SDS-PAGE and verified for mass accuracy using mass spectrometry. Methods were generally as described as in [38]; protein at approximately 20 µM concentration was heated from room temperature to 80°C in the presence or absence of small molecules, including cyclosporins A, C, D, or H (LKT Labs, MN, USA). The cyclophilins were originally prepared in 100% DMSO at 50–100 mM concentration, then diluted to 50 µM for screening, thereby ensuring the final DMSO concentration was less than 5% during the experiment. Ligand binding was detected by monitoring the increase in T_agg_ in the presence of the ligand; and any compound that caused a >2°C increase in T_agg_ were observed to be outside of the range of experimental error. Each compound was tested at least twice.

### Isothermal Calorimetry

All experiments were performed using a VP-ITC microcalorimeter (Microcal, MA, USA), and data analysis was performed utilizing the Origin 7 software. All experiments were conducted at 25°C. Methods were roughly based on those in [67], with modifications as described. Highly pure proteins were dialyzed into ITC buffer (50 mM Hepes pH 8, 0.2 M NaCl), which was also used to dilute ligand stock to the concentrations used for ITC. In order to obtain strong signal for binding isotherms, proteins were used at concentrations ranging from 50 to 300 µM, with 100 µM being standard for most cyclophilins tested. The proteins were loaded into the syringe, with the ligand (cyclosporin A, LKT Labs, MN, USA) in the cell at 5 µM concentration. Generally 5–10 µL injections of protein were made; optimal volumes were determined experimentally to obtain reasonable data for single-site fitting. Ligands were described as not binding protein under these conditions if, at high concentrations of protein (∼300 µM), no change in isotherm deflection was noted after 10–20 injections (275 µL of protein).

### NMR-Based Activity Assay

Most protein samples aimed at assessing binding and/or catalysis of tetrapeptide substrates were diluted to 500 µL with 10% D_2_O and placed into a Shigemi microcell (Allison Park, PA, USA). Typical samples contained 0.075 mM protein and 2 mM of suc-AAPF-pNA, suc-AFPF-pNA, or suc-AGPF-pNA (Bachem), along with 100 mM phosphate buffer pH 7 and 100 mM NaCl. Spectra were collected at 25°C on a Varian 600 or 900 MHz spectrometer (Palo Alto, CA, USA). Spectra were acquired using standard Varian BioPack sequences, processed using NMRpipe software [79] and visualized using CCPN software [80]. For samples used to assess binding of PPIA to peptides DEGPF, DFGPF, DYGPF, YGGPF, or VRGPF, samples were as above except protein concentration was 0.3 mM and spectra were collected at 10°C.

### Monte Carlo Simulations

A set of 400 test peptides of the general form X-Z-Gly-Pro were docked to a subset of cyclophilin isoforms (Protein Data Bank [PDB] codes: PPIA, 1AK4: PPIL2, 1ZKC; PPIC, 2ESL; PPWD1, 2A2N; and NKTR, 2HE9) using ICM software (Molsoft LLC). Monte Carlo simulations were performed to sample conformational space for each combination of cyclophilin isoform and test peptide, allowing flexibility of the tetrapeptide and the sidechains of the gatekeepers at positions comparable to PPIA Thr73, Lys82, and Ala103, and keeping the rest of the protein receptor rigid [64]. The crystal structure of PPWD1 (PDB: 2A2N) was used to determine the initial position of each tetrapeptide in the various cyclophilin isoforms by superimposing the Gly and Pro residues onto the corresponding residues bound to the active site of PPWD1, and the catalytic arginine was repositioned to align with Arg535 of PPWD1. Throughout the Monte Carlo simulations (200,000 iterations), tethers were imposed on the C-terminal Gly and Pro residues, to ensure that the tetrapeptides would remain bound to the active site. For each combination of cyclophilin isoform and tetrapeptide, the lowest-energy complex was chosen as the predicted conformation of the bound complex, and an estimate of the binding energy was calculated using ICM (Molsoft, LLC) [65]. Additionally, the distance between the tetrapeptide and the Cα of the gatekeeper equivalent to PPIA Lys82 was calculated (this residue is located at the far end of the S2 pocket; see Figure 4), to determine how well the docked peptide was predicted to fill the S2 pocket. Peptides derived from simulation data were synthesized without modification by the Core Facility at Tufts University (http://tucf.org/).

### Accession Numbers

PDB codes for the novel cyclophilin structures presented within this manuscript are as follows: 2R99 (PPIE), 2ESL (PPIC), 2HE9 (NKTR), 2GW2 (PPIG), 2HQ6 (SDCCAG-10), 1ZKC (PPIL2), and 2A2N (PPWD1). PDB codes for the previously deposited set of structures used to generate figures and analyzed in the text are: 2CPL (PPIA), 2BIT (PPIF), 1CYN (PPIB), 1QOI (PPIH), 1XWN (PPIL1), and 2OK3 (PPIL3). GenBank accession numbers for the cyclophilins noted in the methods are: BC003026 (PPIA), BC020800 (PPIB), BC002678 (PPIC), BC030707 (PPID), BC008451 (PPIE), BC005020 (PPIF), BC001555 (PPIG), BC003412 (PPIH), BC003048 (PPIL1), BC000022 (PPIL2), BC007693 (PPIL3), BC020986 (PPIL4), BC038716 (PPIL6), NM006267 (RANBP2 - synthetic template), BC015385 (PPWD1), BC167775 (NKTR), and BC012117 (SDCCAG-10).

## Supporting Information

Datapack S1 **Standalone iSee datapack - contains the enhanced version of this article for use offline.** This file can be opened using free software available for download at http://www.molsoft.com/icm_browser.html.
(ICB)

Figure S1 **Characterization of isomerases using an NMR-based tetrapeptide activity assay.** Amide-beta correlations of the Ala within the suc-AGPF-pNA peptide are shown from the 1H-1H TOCSY experimental results. Resonances in black are from peptide in the absence of protein; resonances in red are observed upon addition of the isomerase noted above each panel. If there is acceleration of *cis–trans* isomerization that occurs on the fast NMR time scale—i.e., faster than the chemical shift differences between *cis* and *trans* resonances—then the individual resonances coalesce into a single set of resonances. (A) Wild-type enzymes tested in the presence of commercial substrate suc-AGPF-pNA. PPID and PPIG are two examples of active isomerases, while PPIL2 and SDCCAG-10 are not active under the experimental conditions tested. Notice in the cases of PPIL2, and especially SDCCAG-10, that although the resonances do not coalesce—and therefore there is no significant enhancement of isomerization—the peak centers do shift, indicating that the chemical environment of the peptide is changing upon addition of enzyme. This is defined as binding, but not catalysis, for this protein∶substrate pair. (B) Effects of mutations upon PPIA and PPIL2. Mutation of PPIA Trp121 to tyrosine knocks out enzymatic activity upon suc-AGPF-pNA, while mutation of PPIL2 Tyr289 to histidine confers activity to this previously inactive isomerase. (C) Activity of PPIA against peptides derived from computational data.
(7.11 MB TIF)

Figure S2 **Sequence alignment of the human cyclophilin isomerase domains.** Key structural and catalytic residues discussed in the text are labeled. Alignment was generated using ClustalX [82],[83].
(9.89 MB TIF)

Figure S3 **Sequence-based data for the human cyclophilin isomerase domains.** (A) Phylogenetic tree with domain organization for the 17 annotated members of the cyclophilin family of isomerases. (B) A graphical representation of the motifs found in multidomain cyclophilins. Both figures were generated using the Interactive Tree of Life server [84]. (C) Diagonal table showing the percent sequence similarity between the isomerase domains.
(1.05 MB TIF)

Figure S4 **The modeled effects of the residue identity at position 121 in relation to cyclosporin A binding.** In (A), the experimental structure of a complex between PPIA and cyclosporin A (PDB 2RMA) is shown. The distance between the carbonyl moiety of methylleucine 9 and the indole nitrogen of Trp121 is shown. In (B), Trp121 is shown mutated to histidine. The sidechain is oriented with a preferred rotamer conformation and corresponds to the experimentally observed rotamer found in NKTR, which has a naturally occurring histidine at this position. In (C), Trp121 is shown mutated to a tyrosine. The sidechain is oriented with a preferred rotamer position; the steric clashes with Cζ of Phe60 and the carbonyl group methylleucine 9 are highlighted in this orientation. In PPIL2, which naturally encodes a tyrosine at this position, the rotamer found is oriented such that it avoids these potential steric clashes (see Figure 2).
(2.63 MB TIF)

Figure S5 **Regions of structural diversity in the human cyclophilins.** (A) An overlay of PPIA in blue, PPWD1 in red, PPID in grey, and NKTR in pink are shown. Alignment is global over all atoms, and for all structures is less than 2 Å (1.4 Å for PPWD1, 0.491 Å for PPID, and 0.631 Å for NKTR). Regions of structural diversity are highlighted with labels and zoomed in the panels below. (B) The structure of the β1-β2 loop region is shown for PPIA and PPWD1. (C) The structure of the α1-β3 loop region is shown for PPIA, PPWD1, and NKTR. (D) The structure of the α2-β8 loop is shown for PPIA, PPID, and NKTR.
(3.62 MB TIF)

Figure S6 **Additional results from simulations.** Scatter plots corresponding to the dynamic simulations on NKTR, PPIC, PPIL2, and PPWD1 are shown. Axes and coloring are as in Figure 6.
(2.23 MB TIF)

Table S1 **Crystallographic data and refinement statistics.** ^a^Highest-resolution shell is shown in parentheses. ^b^R_sym_ = 100×sum(| I−< I >|)/sum(< I >), where I is the observed intensity and < I > is the average intensity from multiple observations of symmetry-related reflections. ^c^R_free_ value was calculated with 5% of the data.
(1.81 MB TIF)

Text S1 **Instructions for installation and use of the required Web plugin (to access the online enhanced version of this article).**
(0.75 MB PDF)

Text S2 **Supplemental methods.**
(0.03 MB DOC)

## Abbreviations

CsA: cyclosporin A
ITC: isothermal calorimetry
LIC: ligation-independent cloning
PDB: Protein Data Bank
PPIase: peptidyl-prolyl isomerase
RRM: RNA-recognition motif
TOCSY: total correlation spectroscopy
