# Supplementary figures and images for: Structural and Biochemical Characterization of the Human Cyclophilin Family of Peptidyl-Prolyl Isomerases
Source: PLoS Biol. 2010 Jul 27;8(7):e1000439. doi: 10.1371/journal.pbio.1000439 (PMC2911226; doi:10.1371/journal.pbio.1000439)

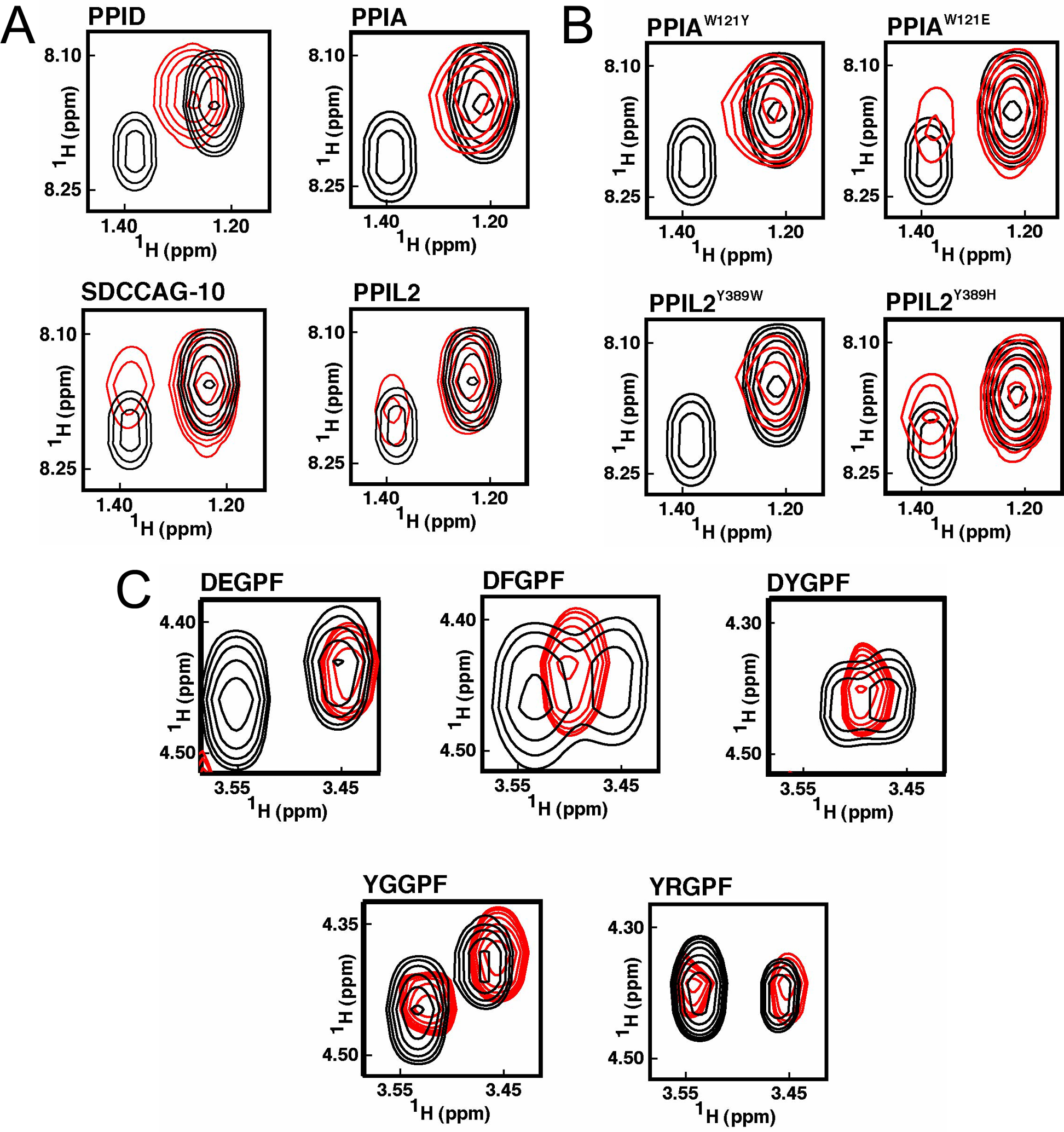

Supplement: Figure S1 — Characterization of isomerases using an NMR-based tetrapeptide activity assay. Amide-beta correlations of the Ala within the suc-AGPF-pNA peptide are shown from the 1H-1H TOCSY experimental results. Resonances in black are from peptide in the absence of protein; resonances in red are observed upon addition of the isomerase noted above each panel. If there is acceleration of cis–trans isomerization that occurs on the fast NMR time scale—i.e., faster than the chemical shift differences between cis and trans resonances—then the individual resonances coalesce into a single set of resonances. (A) Wild-type enzymes tested in the presence of commercial substrate suc-AGPF-pNA. PPID and PPIG are two examples of active isomerases, while PPIL2 and SDCCAG-10 are not active under the experimental conditions tested. Notice in the cases of PPIL2, and especially SDCCAG-10, that although the resonances do not coalesce—and therefore there is no significant enhancement of isomerization—the peak centers do shift, indicating that the chemical environment of the peptide is changing upon addition of enzyme. This is defined as binding, but not catalysis, for this protein∶substrate pair. (B) Effects of mutations upon PPIA and PPIL2. Mutation of PPIA Trp121 to tyrosine knocks out enzymatic activity upon suc-AGPF-pNA, while mutation of PPIL2 Tyr289 to histidine confers activity to this previously inactive isomerase. (C) Activity of PPIA against peptides derived from computational data. (7.11 MB TIF) [file pbio.1000439.s002.tif]

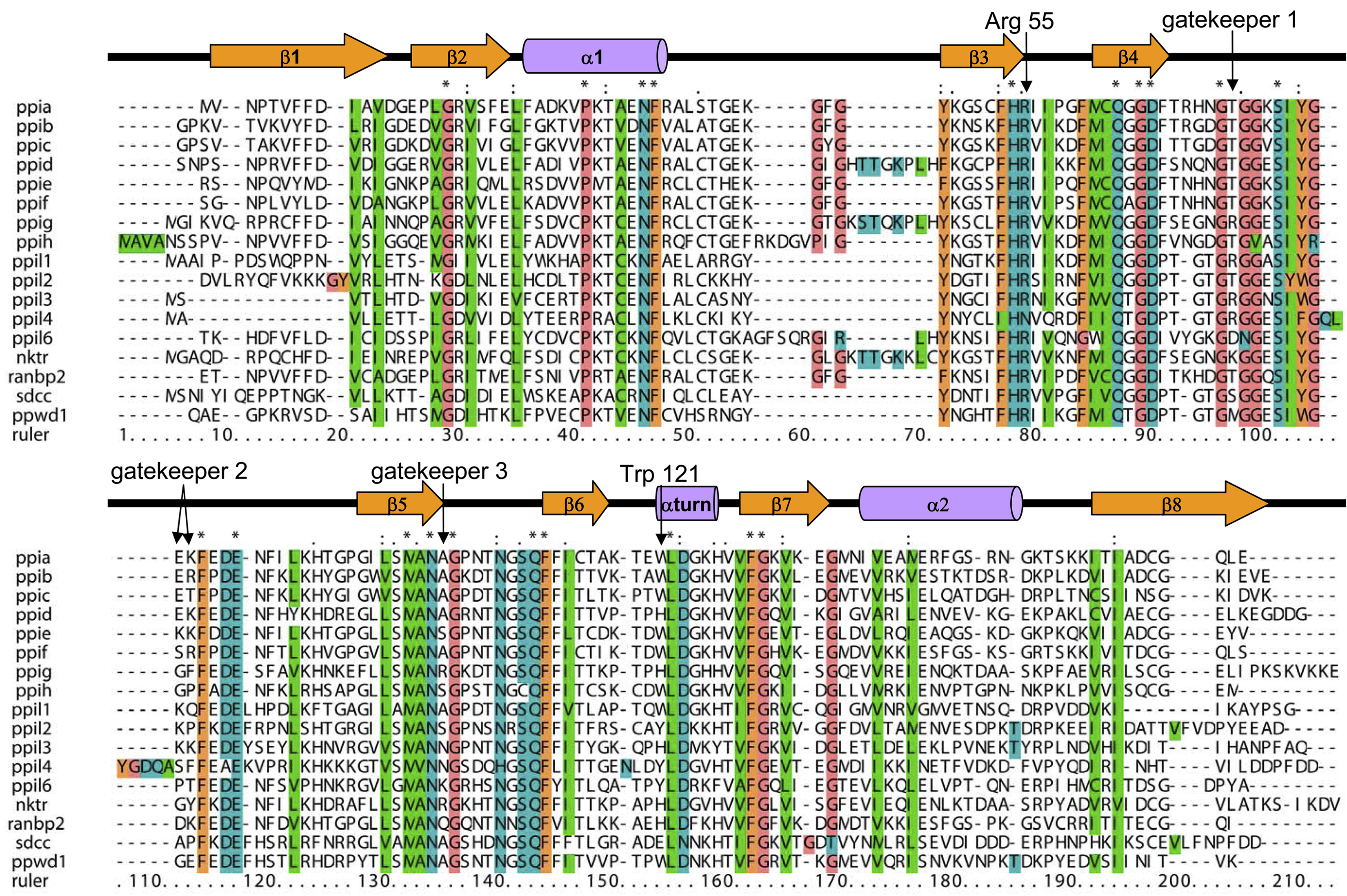

Supplement: Figure S2 — Sequence alignment of the human cyclophilin isomerase domains. Key structural and catalytic residues discussed in the text are labeled. Alignment was generated using ClustalX [82],[83]. (9.89 MB TIF) [file pbio.1000439.s003.tif]

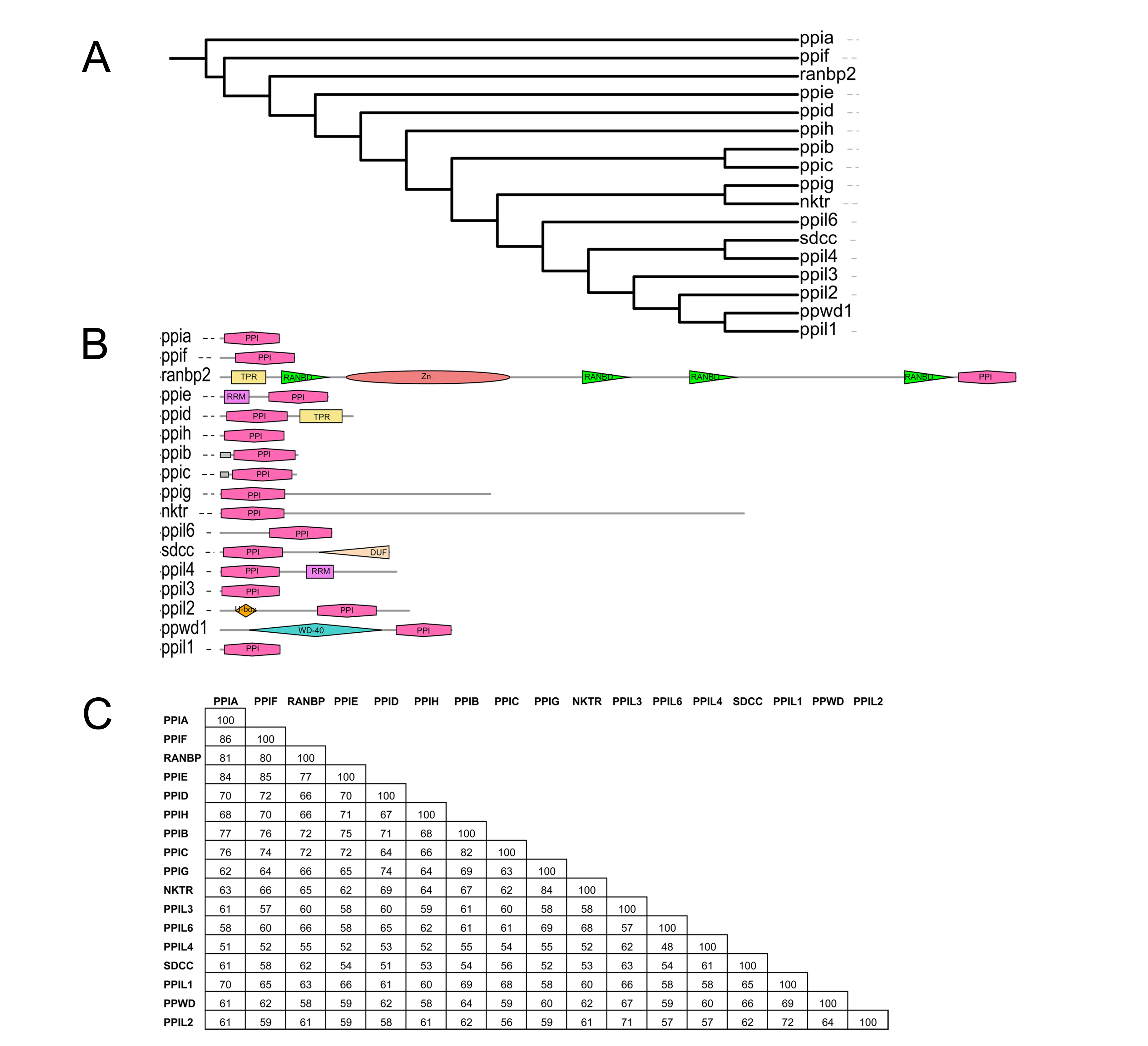

Supplement: Figure S3 — Sequence-based data for the human cyclophilin isomerase domains. (A) Phylogenetic tree with domain organization for the 17 annotated members of the cyclophilin family of isomerases. (B) A graphical representation of the motifs found in multidomain cyclophilins. Both figures were generated using the Interactive Tree of Life server [84]. (C) Diagonal table showing the percent sequence similarity between the isomerase domains. (1.05 MB TIF) [file pbio.1000439.s004.tif]

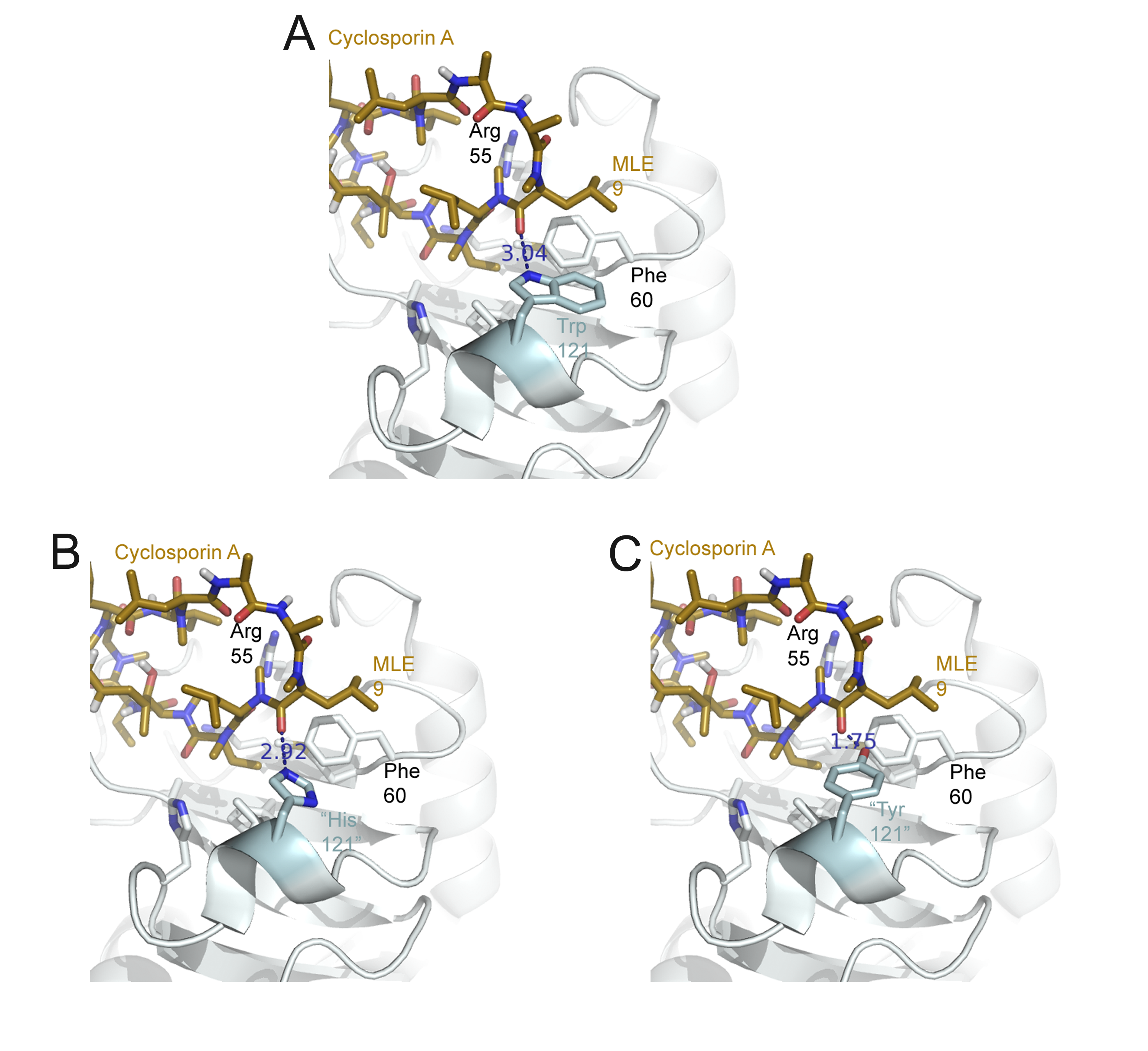

Supplement: Figure S4 — The modeled effects of the residue identity at position 121 in relation to cyclosporin A binding. In (A), the experimental structure of a complex between PPIA and cyclosporin A (PDB 2RMA) is shown. The distance between the carbonyl moiety of methylleucine 9 and the indole nitrogen of Trp121 is shown. In (B), Trp121 is shown mutated to histidine. The sidechain is oriented with a preferred rotamer conformation and corresponds to the experimentally observed rotamer found in NKTR, which has a naturally occurring histidine at this position. In (C), Trp121 is shown mutated to a tyrosine. The sidechain is oriented with a preferred rotamer position; the steric clashes with Cζ of Phe60 and the carbonyl group methylleucine 9 are highlighted in this orientation. In PPIL2, which naturally encodes a tyrosine at this position, the rotamer found is oriented such that it avoids these potential steric clashes (see Figure 2). (2.63 MB TIF) [file pbio.1000439.s005.tif]

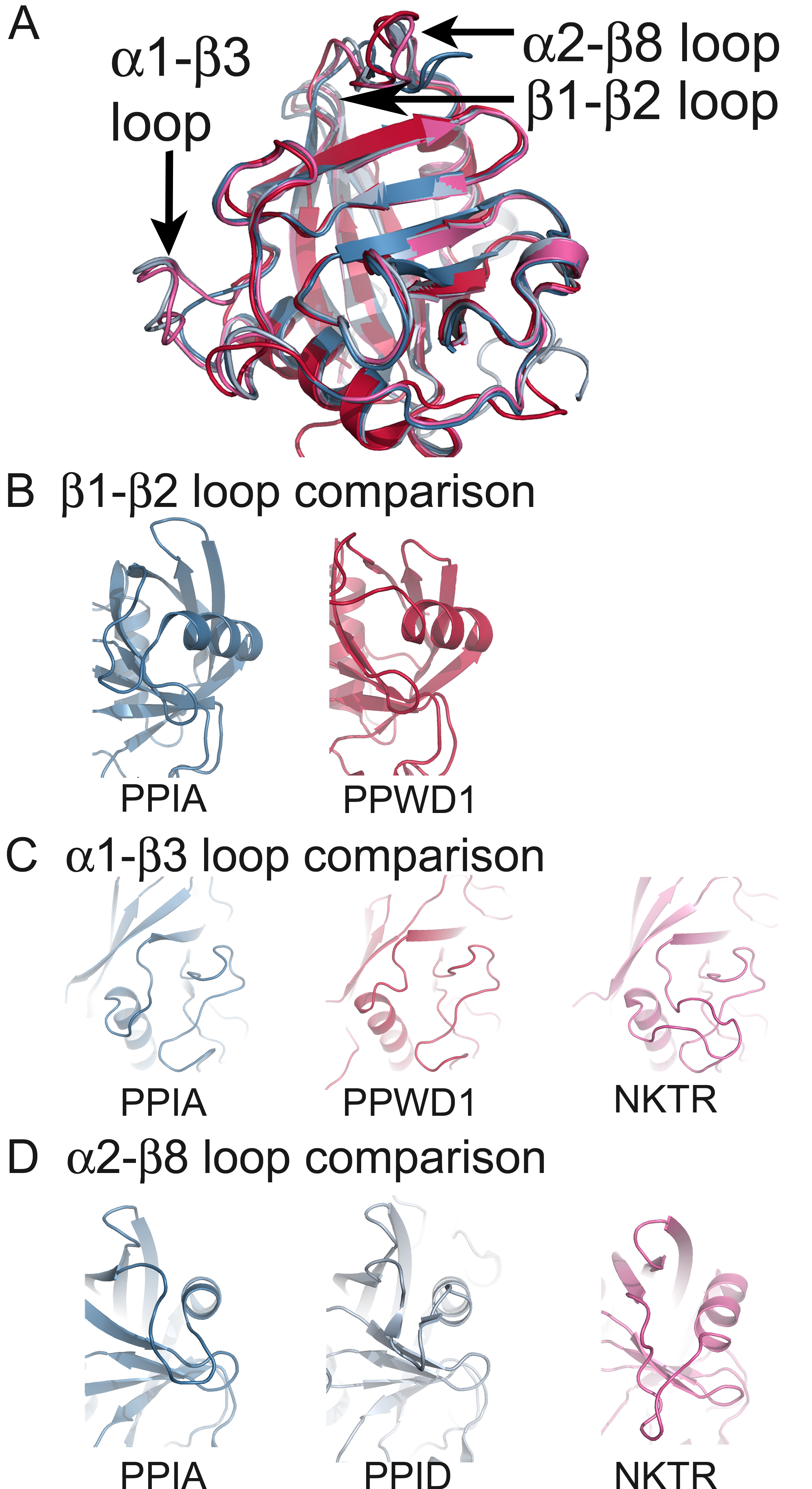

Supplement: Figure S5 — Regions of structural diversity in the human cyclophilins. (A) An overlay of PPIA in blue, PPWD1 in red, PPID in grey, and NKTR in pink are shown. Alignment is global over all atoms, and for all structures is less than 2 Å (1.4 Å for PPWD1, 0.491 Å for PPID, and 0.631 Å for NKTR). Regions of structural diversity are highlighted with labels and zoomed in the panels below. (B) The structure of the β1-β2 loop region is shown for PPIA and PPWD1. (C) The structure of the α1-β3 loop region is shown for PPIA, PPWD1, and NKTR. (D) The structure of the α2-β8 loop is shown for PPIA, PPID, and NKTR. (3.62 MB TIF) [file pbio.1000439.s006.tif]

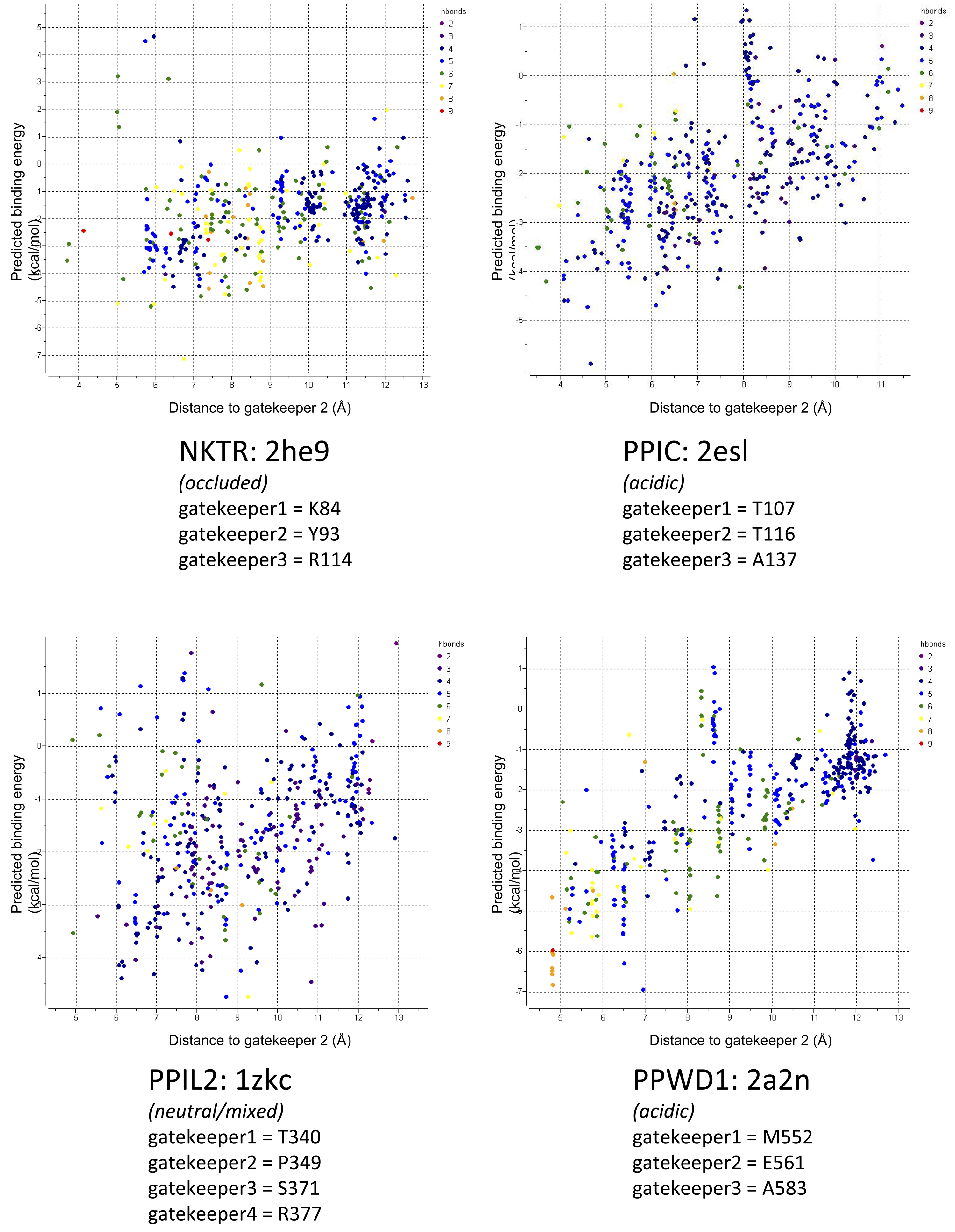

Supplement: Figure S6 — Additional results from simulations. Scatter plots corresponding to the dynamic simulations on NKTR, PPIC, PPIL2, and PPWD1 are shown. Axes and coloring are as in Figure 6. (2.23 MB TIF) [file pbio.1000439.s007.tif]

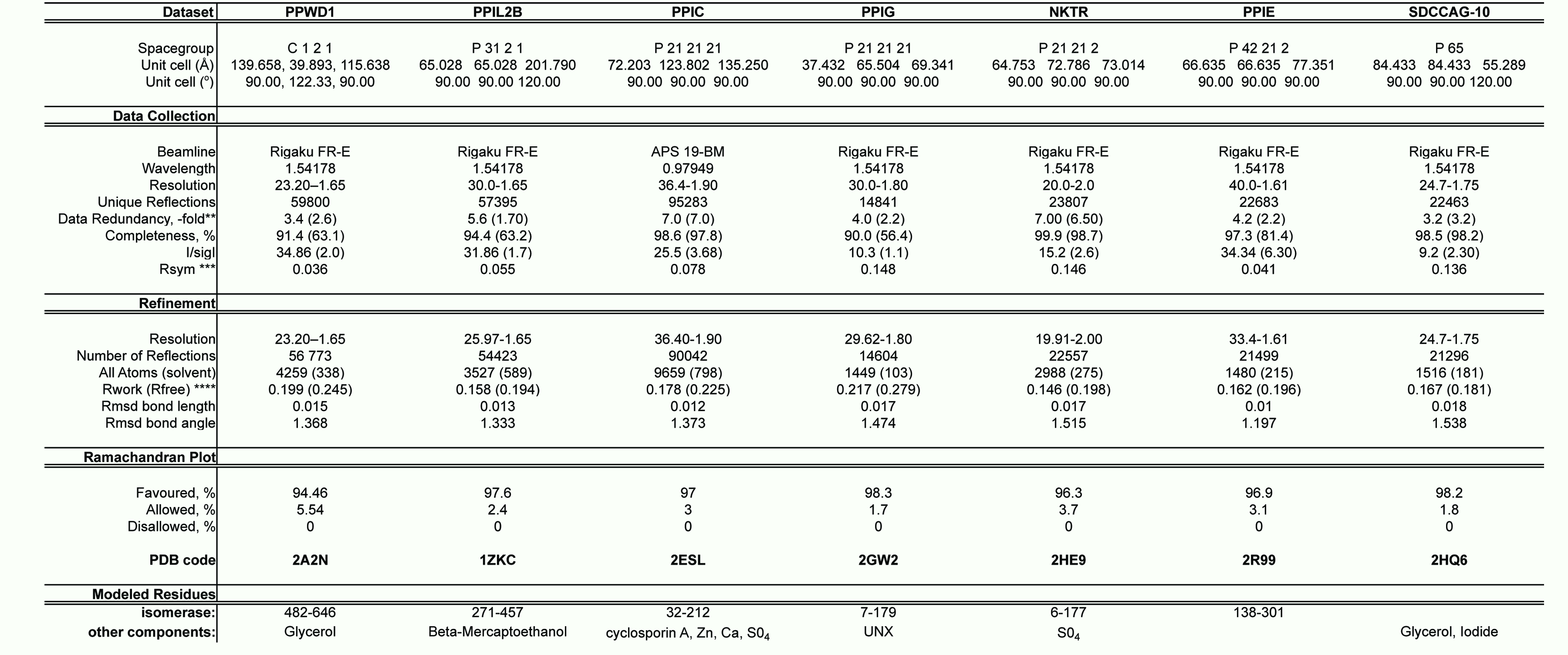

Supplement: Table S1 — Crystallographic data and refinement statistics. aHighest-resolution shell is shown in parentheses. bRsym = 100×sum(| I−< I >|)/sum(< I >), where I is the observed intensity and < I > is the average intensity from multiple observations of symmetry-related reflections. cRfree value was calculated with 5% of the data. (1.81 MB TIF) [file pbio.1000439.s008.tif]
