# Supplementary material for: Structural and Biochemical Characterization of the Human Cyclophilin Family of Peptidyl-Prolyl Isomerases
Source: PLoS Biol. 2010 Jul 27;8(7):e1000439. doi: 10.1371/journal.pbio.1000439 (PMC2911226; doi:10.1371/journal.pbio.1000439)
Supplement: Text S2 — Supplemental methods. (0.03 MB DOC) [file pbio.1000439.s010.doc]

**Text S2.**

**Supplemental methods.**

PPIG protein was mutated using residues predicted by the Surface Entropy Reduction server at <http://nihserver.mbi.ucla.edu/SER/>. Double point mutations K125A and E126A were introduced using the Quickchange kit (Stratagene). The expression construct was transformed into BL21 DE3 Codon Plus RIL cells for growth. Cells were grown to OD_600_ of 1 and then induced with 1 mM IPTG overnight at 15 °C. Cells were harvested by centrifugation at 8,000 rpm. The cell pellets were frozen in liquid nitrogen and stored at −80 °C. For the purification the cell paste was thawed and resuspended in lysis buffer (50 mM Hepes, pH 7.5, 0.5 M NaCl, 5 mM imidazole, 2 mM β-mercaptoethanol, 5% glycerol) with protease inhibitor (1 mM phenylmethyl sulfonyl fluoride). The cells were lysed by passage through a Microfluidizer (Microfluidics Corp.) at 20,000 psi. The crude extract was cleared by centrifugation. The clarified lysate was loaded onto a 3 mL Ni-NTA column (Qiagen). The column was washed with 5 column volumes of 50 mM Hepes buffer, pH 7.5, containing 500 mM NaCl, 5% glycerol and 25 mM imidazole, and the protein was eluted with elution buffer (50 mM Hepes, pH 7.5, 500 mM NaCl, 250 mM imidazole, 5% glycerol). The protein was loaded onto a Superdex 200 column (Amersham Biosciences) equilibrated with 50 mM Hepes buffer, pH 7.5, and 500 mM NaCl, 5% glycerol, 1 mM DTT. Combined fractions were concentrated to 15.5 mg/mL. The purification yield was 55 mg of purified protein per 1 L of culture.
